# Differences between users’ and addiction medicine experts’ harm and benefit assessments of licit and illicit psychoactive drugs: Input for psychoeducation and legalization/restriction debates

**DOI:** 10.3389/fpsyt.2022.1041762

**Published:** 2022-11-16

**Authors:** Udo Bonnet, Michael Specka, Ann-Kristin Kanti, Norbert Scherbaum

**Affiliations:** ^1^Department of Psychiatry, Psychotherapy and Psychosomatic Medicine, Evangelisches Krankenhaus Castrop-Rauxel, Castrop-Rauxel, Germany - Academic Teaching Hospital of the University of Duisburg-Essen, Essen, Germany; ^2^Department of Psychiatry and Psychotherapy, LVR-Hospital Essen, Faculty of Medicine, University of Duisburg-Essen, Essen, Germany; ^3^Department of Internal Medicine, Evangelisches Krankenhaus Castrop-Rauxel, Castrop-Rauxel, Germany - Academic Teaching Hospital of the University of Duisburg-Essen, Essen, Germany

**Keywords:** traditional illicit drugs, cannabis, new psychoactive drugs, ketamine, assessment bias, prescription drugs, pregabalin, gabapentin

## Abstract

**Background:**

There is a lack of benefit/harm assessments of illicit and licit psychoactive substances performed by substance-dependent users in comparison to addiction medicine experts.

**Methods:**

We extended the analyses of substance harm/benefit assessments of German addiction medicine experts (*N* = 101), in parts reported recently in this journal [doi.org/10.3389/fpsyt.2020.59219], by the perspectives of substance-addicted persons. The same questionnaire as used for the abovementioned “experts-study” was handed out to inpatient detoxification or rehab treatment seeking German substance-dependent adults (*N* = 117) for a subsequent structured interview about harms and benefits of 33 new and traditional psychoactive substances comprising also prescription drugs.

**Results and discussion:**

Both, users and experts, ranked the traditional illicit psychoactive substances heroin, cocaine and amphetamines within the top overall harm level group. Synthetic cannabinoids, alcohol and benzodiazepine were in a subordinate top-harm level position. Both cohorts also ranked methadone, nicotine and cannabis within the midrange and buprenorphine as well as psychotropic mushrooms within the lowest harm level positions. Experiences with prescription drugs (including opioidergic analgesics and gabapentinoids), cathinones, GHB, methamphetamine and methylphenidate was not prevalent in our user population. The same applied to barbiturates, propofol, kratom, ayahuasca with nearly zero assessments for each substance. The most user-experiences (>50% per assessed substance) were reported with nicotine, cannabis, alcohol, cocaine, heroin, amphetamine and methadone (core group). The user’s overall harm ratings in terms of these psychoactive substances were similar to those of the experts with the exception of the methadone assessment which was rated by the experts to be significantly less harmful if compared with the users’ estimation (supposed “treatment bias” of experts). The users’ benefit ratings for the traditional illicit psychoactive substances, cannabis as well as for nicotine were significantly more positive in comparison to those of the experts (supposed “attraction bias” of users). Both, experts and users, ranked the harms arising from the use of alcohol or benzodiazepines (usually unregulated substances) higher than the harms caused by the use of methadone, cannabis or psychotropic mushrooms (regulated by most Western narcotic acts). Users attributed the most benefits to buprenorphine, methadone and cannabis. This might reflect a main limitation of the study as the data are from an user population comprising over 50% patients who sought detoxification-treatment of opiates where methadone and buprenorphine are usual transient medications (supposed “selection bias”).

**Conclusion:**

This study addressed current trends of psychoactive substance abuse (e.g., synthetic cannabinoids, prescription drugs) and provides from both perspectives (that of the user and that of the addiction medicine experts) robust harm/benefit evaluations at least of a core group of psychoactive substances (traditional illicit psychoactive substances, cannabis, methadone, alcohol and nicotine). The results of this study can be valuable to the psychoeducation of substance-addicted individuals and to current restriction/legalization debates, especially in the Western-EU.

## Introduction

Persons who are using psychoactive substances have a lot of motives in doing so besides medical-therapeutic reasons. Non-medical motives comprise, e.g., self-medication, recreational, mind-altering, spiritual, affect-regulating, relaxing, doping, cognition-enhancing or sleep-improving purposes. As a rule, those aims are perceived as “beneficial.” However, the most psychoactive substances are also “hijacking” the mesolimbic dopaminergic reward system as a biological condition *sine qua non for* burgeoning and maintaining addictive behavior (1–4). The rewarding vigor of an individual psychoactive substance, as well as its availability, the context cues and the user’s vulnerability are powerful predictors of a substance abuse and dependence (ICD-10, DSM-IV TR III) or substance use-disorder (SUD) (3–5). Severely affected persons are compulsively using multiple psychoactive substances for non-medical purposes, displaying substance misuse spiraling out of executive self-control (4, 5). They invest a lot of their lifetime to get these substances to maintain pleasant intoxication effects (e.g., feeling “high,” relaxed or energetic) and to avoid physical and behavioral withdrawal; in sum this often leads to personal and social depravation; and in the case of illicit psychoactive substance use, more probably to delinquent behavior and substance related deaths (3–5).

Nutt et al. developed a consensus-based rating instrument for assessing an individual psychoactive substance’s overall harm potential considering physical, psychological and social harms to users and others (6). Within the last 20 years, several ratings by experts in the area of addictive disorders were carried out in several Western European countries and Australia (1, 6–12). These rankings do not necessarily show congruence with legislative and law enforcement priorities in terms of regulation and control of substances, with alcohol being a classic example of dissonance between a high level of overall harm and low levels of governmental regulation (1, 6–13). Few expert rankings also included potential beneficial effects of psychoactive substances (9, 14); and only one study included several prescription drugs being currently debated to be addictive, such as Non-Steroidal Anti-Inflammatory drugs (NSAIDs) and gabapentinoids (GPTs) (1, 14).

As of September 2022, there are very few comparative rankings of perceived harms and benefits arising from the use of psychoactive substances, which have been carried out by substance users (15, 16). However, these are essential to understand the relevance of the experts’ rankings for naturalistic treatment conditions and psychoeducation approaches. Therefore, we conducted a cross-sectional study including adult Germans undergoing an inpatient drug detoxification or inpatient drug users’ rehabilitation treatment. The results from the users’ perspective were compared with the results of the ranking of German addiction medicine experts, which have been recently published (1, 14). Users’ and experts’ rankings were based upon data collected within 2016 to 2019 in Germany and therefore, should reflect some regional as well common trends, including the emergence of novel psychoactive substances, such as synthetic cannabinoids and cathinones (17, 18), see also Supplementary material 2.1), or the increasing misuse of gabapentinoids (1, 19) and further prescription analgesics (20). We hypothesize that also in our study the rankings of both, experts and users, were not congruent with the narcotics acts of Western nations, at least for the position of alcohol. Furthermore, the results might be of interest for current restriction debates (e.g., domestic and abroad for gabapentinoids and alcohol) or legalization debates (e.g., for cannabis or psychotropic mushrooms) (21–23).

## Materials and methods

The present cross-sectional study comprised two consecutive steps (survey 1 and survey 2, see below), in which quantitative questionnaires were distributed for a structured interview among German adults affected by substance dependence (ICD-10) and being admitted either to inpatient detoxification treatment (Evangelisches Krankenhaus Castrop-Rauxel) or inpatient rehab treatment (salus Klinik Castrop-Rauxel). Both hospitals are located close to each other, within the metropolitan Ruhr Area (see Supplementary material 2.1), Germany. The concept of this study (spanning from March 2017 to January 2019, Supplementary Figure 2) is the similar to our previous study (surveys carried out from March 2016 to May 2018, Supplementary Figure 2), which included addiction medicine experts recruited at German addiction congresses and conferences (1, 14). The present study, in contrast, additionally includes users of psychoactive substances, in order to enable a comparison between users’ and experts’ perspectives. The final results are for each group are based on two surveys: the first collected harms and benefits ratings, using pre-defined categories, and the second survey collected relative weights of the criteria, in order to calculate overall harm ratings (Supplementary Figure 1).

### Expert ratings

**FIGURE 1:**
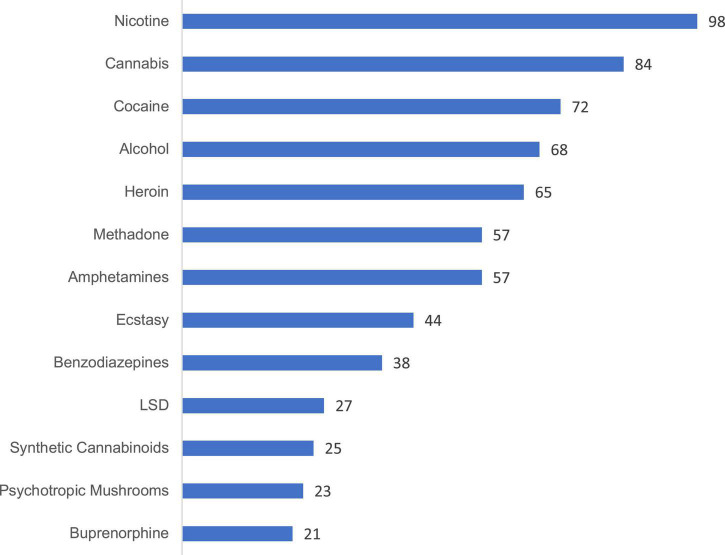
Number of ratings made by user cohort 1, per substance. The minimum number of ratings required for inclusion of a substance was determined as *n* = 20. Extended version in Supplementary Figure 15.

The results of the expert survey were reported elsewhere (1, 14). Inclusion criteria were: to be a physician who (i) was a specialist, i.e., had extra expertise in at least one medical specialty and (ii) had been working longer than 5 years in German hospitals in the area of substance use disorders (SUD) treatment. The *first experts’ survey* was conducted from March 2016 to September 2017 (Supplementary Figure 2) and assessed the average harm of 33 substances (Figure 1) in 5 dimensions (physical harm to users, psychological harm to users, social harm to users, physical and psychological harm to others, and social harm to others). As shown in Supplementary Figure 1, these dimensions were defined by 16 criteria, which have been validated in several studies of this type (6, 8, 12) (see Supplementary material 2: “Methods”). Overall harm to users and overall harm to others comprised three (physical, psychological, social) dimensions and two (physical & psychological, social) dimensions, respectively (for details see Supplementary Figure 1). The assessments were carried out using 5-point scales (from “not harmful” to “extremely harmful”). Furthermore, overall beneficial substance effects were rated per 3-point scales (“no/little”/“moderate”/“a lot”).

The expert cohort comprised 101 participants. The physicians were advised to decide for themselves whether to assess a substance or not, and they were instructed to estimate their professional experience (“no/little”/“moderate”/“a lot”) with each substance they had rated. This information was used to determine the validity of the ratings and to verify defined exclusion criteria. In this context, a substance with less than 60% ratings was excluded from further analysis. Thus, the substances ayahuasca, khat, and kratom had to be excluded from the experts’ harm and benefit evaluation (1, 14). The *second experts’ survey* (for determining the relative weight of each health and social harm dimension to calculate the overall harm of each assessed substance, see Supplementary Figure 1) was conducted from September 2017 to May 2018 by cohort 2 (Supplementary Figure 2), which were recruited from the emails to 40 heads of German substance addiction treatment centers. This survey was separate because the first survey was very comprehensive; and combining the two surveys was deemed likely to reduce the return rate of cohort 1. All 36 returned questionnaires could be included. We used the mean relative weight given by the 36 experts to each dimension for calculating the overall harm of each substance (Table 1).

**TABLE 1:** Relative weights of harm dimensions, as determined by German addiction experts, patients, and EU experts.

Dimension	EU-rating* (Experts, *n* = 40)	Experts** (*n* = 36)	Patients (user)** (*n* = 44)
		Mean (SD) in %	Mean (SD) in%
Physical harm to user	25.9	25.0 (7.0)	21.3 (9.6)
Psychological harm to user	16.0	23.5 (6.6)	28.3 (13.2)
Social harm to user	11.2	20.1 (7.5)	23.9 (12.6)
Physical & psychological harm to others	11.6	13.0 (4.4)	14.1 (7.4)
Social harm to others	35.5	18.3 (7.7)	12.3 (7.5)

Weights add up to 100%. *Consensus-based (8), **ad hoc, clearly different estimations are marked by gray background. The calculation of the overall harm of the substances by using these relative weights are shown in the Supplementary material 2.2.

### User ratings

The same questionnaires which had been developed for the experts’ rating of substance-related overall harm and benefit were handed out to the users during their inpatient treatment period in preparation for a subsequent structured interview (by AK-K). The study was carried out in accordance with the guidelines of the Declaration of Helsinki and was approved by the local ethics committee of the medical faculty of the University of Duisburg-Essen, Essen, Germany. Data collection was from March 2017 to January 2019 (Supplementary Figure 2). All adult patients voluntarily admitted to the ward specialized for detoxification treatments (Evangelisches Krankenhaus Castrop-Rauxel) or to the substance addiction rehab ward of the salus Klinik Castrop-Rauxel during the study period were considered for inclusion.

*Exclusion criteria* were insufficient proficiency of the German language and cognitive impairment (Mini Mental State [MMST] < 25 points). Patents *eligible* for the study who had given informed consent were asked to complete a standardized interview dealing basic sociodemographic data, history of substance use, and assessments of he several psychoactive substances (*first users’ survey*).

Data collection *for cohort 1* was stopped after 100 interviews had been completed. To achieve this sample size, 117 patients had to be screened. Of these, 6 refused to participate, 3 withdrew from the interview after having given informed consent, and 8 had a MMST level <25 points.

The *second users’ survey* for weighting the dimensions to determine the overall harm was conducted within the same pool of inpatients during the aforementioned study period (from November 2018 to January 2019, Supplementary Figure 2) and included the first 44 completed returns of the questionnaires (*cohort 2*) distributed in this context (Table 1).

### Miscellaneous

The users’ and experts’ identity was kept anonymous with the exception of information about their age, gender, specialties (experts), years of professional experience (experts), years of work in secondary/tertiary care (experts) of SUD, and main focus of professional work (acute care or rehabilitation hospital, experts) as well as, addiction history (user), comorbidity, socio-biography (user), respectively (Table 1). Just as for the expert raters (1, 14) we used a ratio of 75:25% for user raters recruited from an acute (EVK Castrop-Rauxel) versus a rehab clinic (salus Klinik Castrop-Rauxel, see also Supplementary material 2.1).

### Data analysis and statistics

**FIGURE 2:**
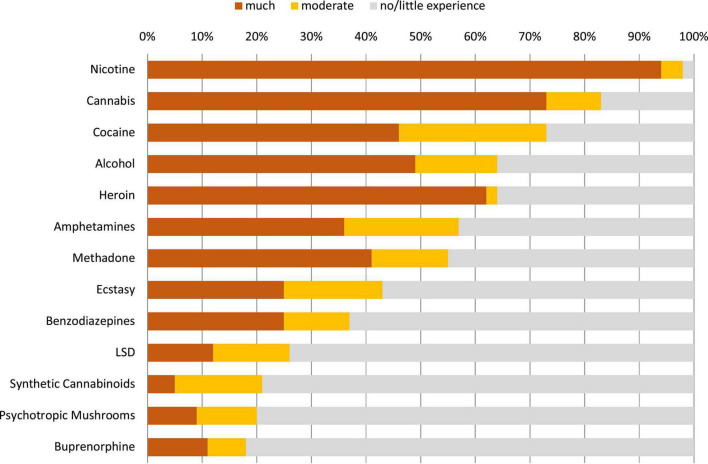
Users’ experience regarding the evaluated substances in cohort 1. The “core group” (defined by <50% “no/little”-experience data and >50% ratings per substance) comprised the substances nicotine, cannabis, cocaine, alcohol, heroin, methadone, and amphetamines. Extended version in Supplementary Figure 16.

We set a *cut-off of n* = 20 for the minimum number of users’ ratings required for a substance in order to be included in the further analysis. With a median standard deviation of about 0.8 for the mean total harm ratings, this cutoff value is associated with a 95% confidence interval around the mean of ±0.35 (which becomes smaller with larger subsample sizes). Below the cutoff were triptanes (0 ratings), barbiturates (0), khat (0), propofol (1), cathinones (1), NSAIDs (1), flupirtine (1), ayahuasca (2, kratom (3), GHB (3), Z-drugs (3), natural hallucinogenics (5), methamphetamine (6), methylphenydate (7), opioidergic analgesics (7), ketamine (9), codeine (11), tilidine/tramadol (11), crack (12), and gabapentinoids (13 ratings) (Supplementary Figure 15). Figure 1 shows the number of ratings per substance (above the cut-off-level) performed by user cohort 1. We also explored a *“core group”* of psychoactive substances with more than 50% user ratings and less than 50% “no/less user experience-ratings for stronger data validation (Figures 1, 2). A *further sensitivity test* was carried out by comparing the overall harm-results when the relative harm dimension weights of the EU-rating (Table 1) were used.

Beyond descriptive *statistics*, group comparisons were carried out by using the Welch-corrected t-test or the Mann–Whitney *U*-test (SPSS version 27) Significance level was set at 0.01.

## Results

### Samples

The experts’ sample characterization is already reported elsewhere (1). The users’ sociodemographic sample characteristics are shown in Table 2 The most frequent substances for which detoxification was sought were opioids (57%: heroin, 47%; opiate replacement therapy/medication, 10%), cannabis (40%), cocaine (31%), benzodiazepines (24%), and alcohol (24%). More than one substance was indicated in 79% of participants. There was no patient who sought a detoxification treatment for prescription analgesics including gabapentinoids (24). Psychiatric or somatic comorbidity was described in 85% of all participants (mostly depression, 36%; hepatitis C, 27%; COPD, 20%).

**TABLE 2:** User characteristics.

	Cohort 1 (*n* = 100)	Cohort 2 (*n* = 44)
**Treatment facility**		
In-patient detoxification	75%	75%
In-patient rehabilitation	25%	25%
**Gender**		
Male	75%	88.6%
Female	25%	11.4%
**Age (mean, SD)**	35.9 (10.4)	38.5 (10.8)
**Nationality**		
German	80%	100%
**Marital status**		
Single	76%	
Firm partnership	4%	
Offspring (yes)	31%	
**Housing situation**		
Alone	51%	
With partner	15%	
With offspring	3%	
With parents	19%	
**School qualification**		
None	27%	
Secondary general school certificate	37%	
High-school diploma (“Abitur”)	11%	
**Vocational qualification**		
No specialist job training	58%	
University degree	1%	
**Professional activity**		
Unemployed	75%	
**Net income (month)**		
<500€	58%	
500–1,000€	16%	
1,000–2,500€	21%	
**Comorbidity**		
None	15%	47.7%
Depression	20%	34.1%
Anxiety/PTSD	8%	13.6%
Schizophrenia	4%	2.3%
Hepatitis C	27%	
COPD	20%	
**Years since start of substance use (mean, SD)**	19 (10)	20 (9)
**Primary substances for detoxification (multiple entries possible)**		
Heroin	47%	59.1%
Opioid replacement therapy/medication (opioid substitutes)	10%	11.4%
Cocaine	31%	34.1%
Alcohol	24%	18.2%
Cannabis	40%	18.2%
Amphetamines	22%	13.7%
Benzodiazepines	24%	29.5%
Other	11%	4.6%

### Average overall harm

**FIGURE 3:**
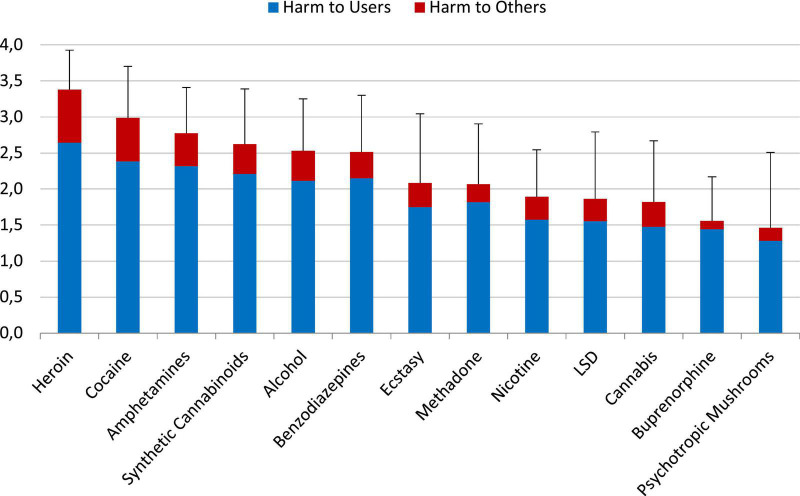
Average overall harm of the evaluated 13 substances (mean values and standard deviations) as assessed by the user cohort 1 on a scale from 0 (“not harmful”) to 4 (“extremely harmful”), shown as harmful to users and harmful to others. The relative contribution of the 5 dimensions (Supplementary Figure 1; Table 1) had been weighted by user cohort 2. Especially valid were the ranks of the *core group* of substances: nicotine, cannabis, cocaine, alcohol, heroin, methadone, and amphetamines (see Data analysis and statistics). Supplementary Figure 17 shows also the results for the excluded substances.

The experts’ overall harm assessments were already reported elsewhere (1). The users’ ratings considering the 5 separate dimensions are shown in the Supplementary Figures 3-7). The overall harm potential of the traditional substances of abuse, i.e., heroin, cocaine and amphetamines, was assessed by the user group to be the strongest of all 13 evaluated substances (Figure 3). Synthetic cannabinoids, alcohol and benzodiazepines had subordinate positions in the top harm-level group. Ecstasy, methadone (preferred in Germany for maintenance therapy of opioid dependence), nicotine, LSD and cannabis had been placed into the midrange. Buprenorphine (in Germany also frequently used for maintenance therapy of opioid dependence) and psychotropic mushrooms fell into the lowest harm ranges (Figure 3).

### Sensitivity analysis: Changes of the rank order by using the EU-weights?

**FIGURE 4:**
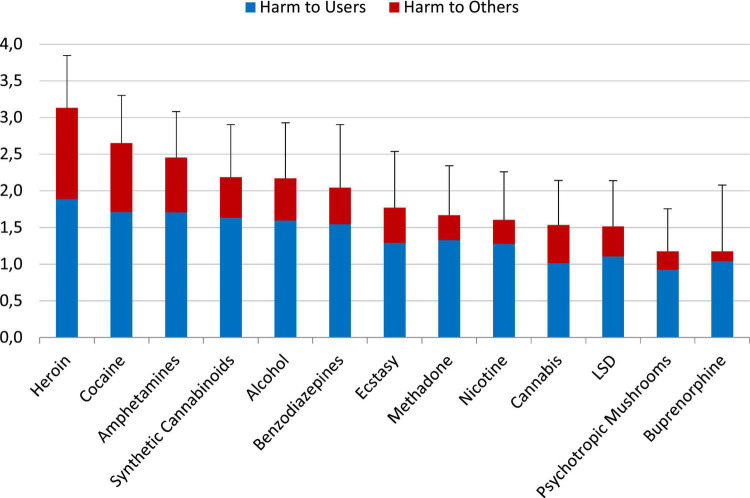
Mean (SD) of the overall harm of the 13 substances using the dimension-weights of the EU-rating (8) on a scale from 0 “not harmful” to 4 “extremely harmful”. Supplementary Figure 18 shows also the results for the excluded substances.

Using the weights determined per an evaluation of Western EU addiction experts (Table 1) did not change the rank order of overall substance harms rated by the experts (1, 14) or by the users in the present study (Figure 4) in a relevant manner.

### Validity of the users’ overall harm-assessments

**FIGURE 5:**
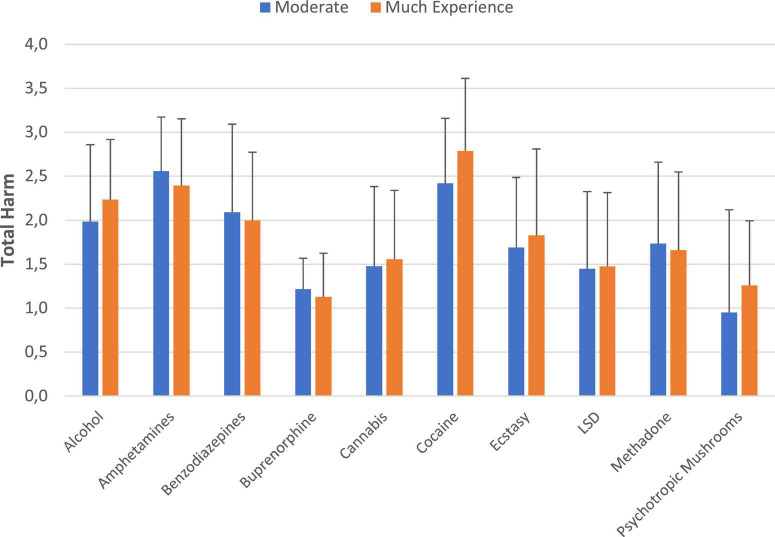
Substance harm-assessments and level of the users’ experience with the corresponding substances. Displayed are substances with *n* > 5 assessments in each experience group. *p < 0.01. The underlying data are shown in Supplementary Table 1.

Figure 5 demonstrates the relationship of the substance harm assessments and the users experience with the corresponding substances. There were no statistical significant differences between the “moderate” and “a lot of” (much) experiences and the average overall harm ratings of each corresponding substance (Figure 5).

### Differences between the user ratings of inpatients treated in an acute and rehab hospital?

Supplementary Figure 8 comprised all substances with more than 6 ratings per substance and setting. We found no statistic relevant differences between the overall harm ratings of the users treated in the acute detoxification unit and the rehab clinic.

### Comparison between overall harm ratings of user and experts

**FIGURE 6:**
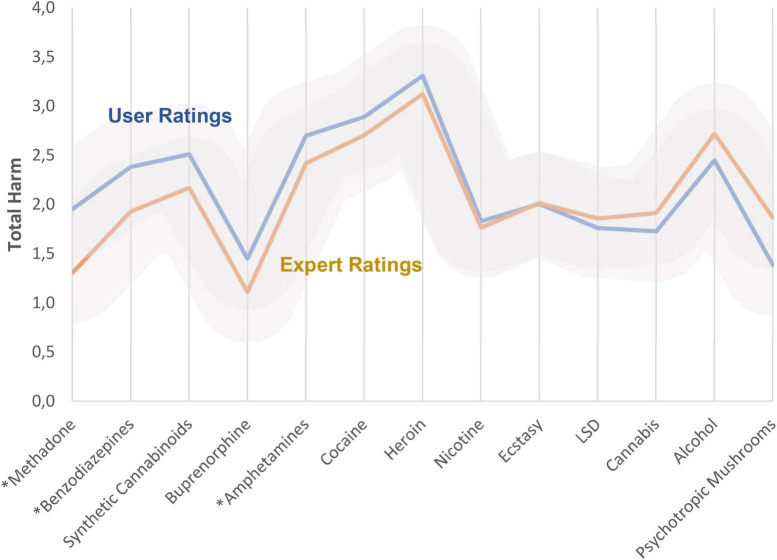
Comparison between users’ and experts’ average overall harm ratings. The relative contribution of the 5 dimensions (Supplementary Figure 1 and Supplementary Table 1) had been weighted by the cohorts 2 of the user and experts. See also Supplementary Figure 20, which demonstrates the rank order. Additionally, the Supplementary Figure 19 shows the ratings for the excluded substances. *p < 0.01. Gray areas indicate standard deviations for the subgroups.

Figure 6 shows this comparison; the underlying ratings considering the separate dimensions are presented in Supplementary Figures 9–13. Both, users and experts ranked traditional illicit psychoactive substances (heroin, crack/cocaine, and amphetamines) within top harm-level positions (Figure 6). The rankings of both groups were very similar. Explicitly, the average overall harm of cocaine, heroin, nicotine, cannabis and alcohol was assessed to be not significantly different between user and experts. However, there were a few exceptions: within the core group, methadone, benzodiazepines and amphetamines are assessed to be significantly more harmful by the users.

### Average overall benefit/utility

**FIGURE 7:**
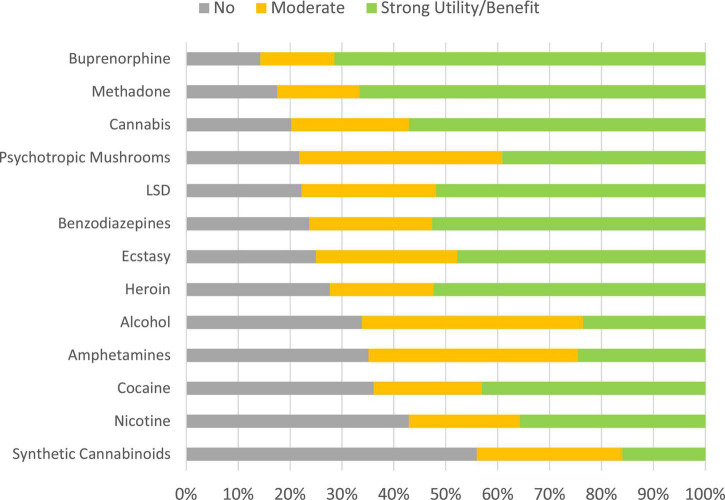
Distributions of benefits categories (cohort 1). The substances are ranked according to proportions of “no benefit” ratings. Supplementary Figure 21 shows also the ratings for the excluded substances.

The experts’ overall benefit assessments are already reported elsewhere (14). Figure 7 shows the user assessments. The strongest benefits/utilities were attributed to methadone, buprenorphine, and cannabis by the users. Synthetic cannabinoids were rated to have the smallest benefits (Figure 7).

### Comparison between overall benefit ratings of users and experts

**FIGURE 8:**
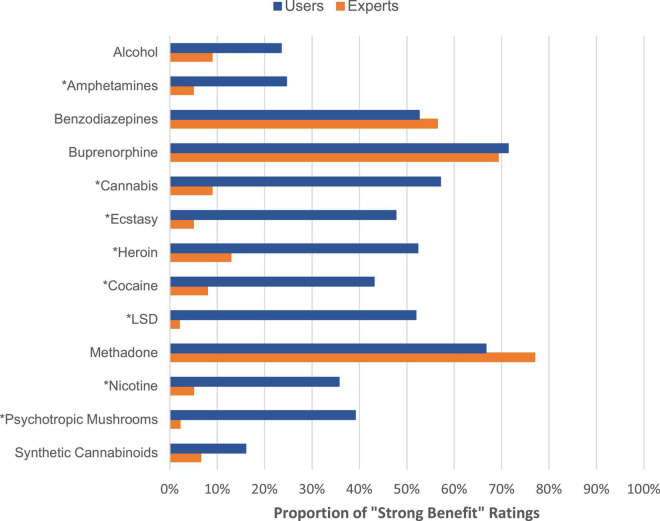
Proportion of “strong benefit” ratings made by users versus experts. *p < 0.01. Supplementary Figure 23 shows also the ratings for the excluded substances.

Figure 8 (proportion of “strong benefit” ratings) and Supplementary Figure 14 (proportion of “no”/”little benefit” ratings) show this comparison. Within the core group, amphetamines, cocaine, heroin, nicotine and cannabis are assessed to be significantly more beneficial by the users. The average overall benefit rating of methadone and alcohol was not significantly different between user and experts. Beyond the core group, further substances were rated to be significantly more beneficial in comparison to the experts: ecstasy, LSD and psychotropic mushrooms.

### Comparison of the overall harm/benefit rating-relations between user and experts

**FIGURE 9:**
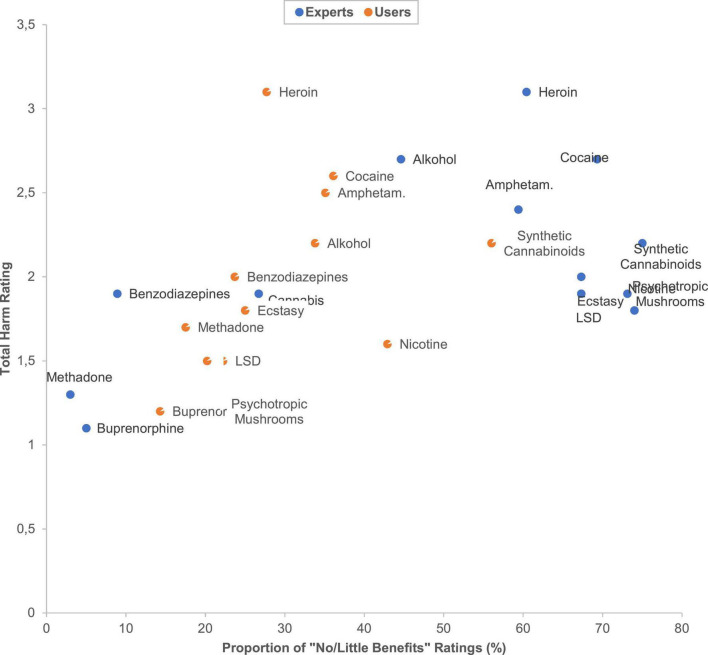
Scatterplot of the users’ and experts’ overall substance harm/benefit ratios. The underlying data are shown in Supplementary Table 2. The users’ cannabis rating point is masked by the users’ LSD rating point. The experts’ nicotine rating point is masked by the experts’ psychotropic mushrooms rating point.

Figure 9 shows these relations. This figure uncovers that the users did not rank any substance in the category with strong harms and less benefits (square top right in this figure). Experts ranked methadone and prescription drugs (opioid analgesics, benzodiazepines, gabapentinoids) to be more beneficial in comparison to users.

## Discussion

To our knowledge, this is one of the first studies (16) comparing the harms and benefits of various psychoactive substances between the assessments of user and experts. Both cohorts ranked the traditional illicit psychoactive substances heroin, cocaine and amphetamines within the top overall harm level group. Synthetic cannabinoids, alcohol and benzodiazepine were in a subordinate top-harm level position. Both, users and experts, ranked methadone, nicotine and cannabis within the midrange and buprenorphine and psychotropic mushrooms within the lowest harm level positions. The Supplementary material shows that ketamine and gabapentinoids belong also to this category; unfortunately the ratings for these substances were below the cut-off of 20 ratings per substance. However, these results might be of interest for current restriction debates according to gabapentinoids (19, 25). As found within previous studies (6, 8, 9, 12, 16), also in our study, the rankings were not congruent with the narcotics acts of Western nations, because both, users and experts, ranked the harms arising from the use of alcohol or benzodiazepines (usually unregulated substances) higher than the harms caused by the use of methadone, cannabis or psychotropic mushrooms, i.e., substances being regulated by most Western narcotics acts.

### Comparison with previous studies

The “backbone” of this rank order is similar to those determined by the most previous substance harm ratings with the following most apparent exception: alcohol was assessed to hold the largest overall harm level according to a majority of expert studies (6, 8, 9, 12) as well as a comparative experts-versus-user study (16). The other available user study placed alcohol in a near, but subordinate position to traditional illicit psychoactive substances, which is in line with the present study, but gave cannabis the lowest harm level position (15), just as the other available user study (16). Most expert evaluations ranked cannabis in a midrange harm position posterior to nicotine/tobacco (6, 8, 9, 12), just as the present study. The user group of the present study assigned methadone and buprenorphine to produce the largest beneficial effects. This assessment differed from prior user evaluations which preferred alcohol, tobacco and cannabis (9, 15, 16). We assume, that this disagreement most likely resulted from the special features of our user population comprising over 50% patients who sought detoxification-treatment of opiates where methadone and buprenorphine are usual transient medications (*selection bias*).

### Further experiences and biases

Non-medical use of prescription drugs was not prevalent in our adult population seeking drug detoxification treatment. The same applied to barbiturates, propofol, kratom, ayahuasca with nearly zero assessments for each psychoactive substance. For non-opioidergic prescription drugs, the most user-experiences were reported for gabapentinoids (7%, see Supplementary Figure 16). 11, 9, and 6% of the patients reported experiences with tilidine/tramadol, codeine and opioidergic analgesics, respectively (Supplementary Figure 16). This relatively low experience level with opioidergic prescription drugs was in accordance with the situation in Germany, not suffering from an opioid epidemic which is contrasting with the situation in other Western nations (25–27). For the remaining substances, the most user-experiences (>50%) were reported with nicotine, cannabis, alcohol, cocaine, heroin, amphetamine and methadone (core group). The user’s overall harm ratings in terms of these core-group psychoactive substances were similar to those of the experts with the exception of the methadone assessment which was rated by the experts to be significantly less harmful if compared with the users’ estimation (availability bias by experts’ positive treatment and mortality-risk-reducing experiences with methadone in opioid addicts (28): *“treatment bias”*).

Notably, the users’ benefit ratings for the traditional illicit psychoactive substances heroin, cocaine, cannabis as well as for nicotine were significantly more positive in comparison with those of the experts. This phenomenon becomes especially obvious if we look at the missing user-assignments of substances in the category of substances with less benefit and great harm in Figure 9, which is clearly contrasting to the experts assessments. We assumed an underlying specific “user bias” resulting from a hedonic imbalance of the dopaminergic mesolimbic reward system (2) due to past addictive substance use experiences (“attraction bias”; resembling the “valence or hedonic tone bias” of emotions). This special bias of addicted persons might foster their substance craving and affective decision-making to relapse. In this regard, a large body of investigations supported a special implicit cognition of addicted persons, acquired by repetitive use of addictive substances or behaviors. Modern addiction theories are based upon this knowledge. In this context, it is hypothesized that repetitive rewarding/reinforcing substance use mediates (i) a sensitization of frontolimbic networks through which substance-related stimuli become emotionally/motivationally salient (attention bias; *“attraction bias”*) as well as (ii) an inhibition of cognitive control functions (both in the sense of addiction-related specific executive cognitive dysfunctions) (29–31). The modulation of these cognitive dysfunctions/biases is regarded as key challenge in the treatment of substance addiction (32).

### Limitations and strengths

The representativeness of the users’ assessments is narrowed to a population of inpatient drug detoxification/rehab seeking adults of a Western German metropole region. The robustness of the experts’ and users’ overall harm rankings after stressing the results by sensitivity analyses (with EU-weights) might at least partially ameliorate this problem. Nonetheless, the compliance deficit of users (great portion of non-responses to substance-specific questions) and the corresponding exclusion of hazardous psychoactive substances, such as methamphetamine, which is considerably more stronger distributed, e.g., in the Eastern EU and Eastern provinces of Germany (1), consists to be a major limitation of this study. Similarly, users and experts from populations who are/were affected by an opioid epidemic most likely might assess the harm/benefit ratio of prescription opioids and gabapentinoids clearly greater than our participants residing in a region without opioid epidemic experiences (25), although about 50% of the participating users had wanted a detoxification of opioids (heroin and opioid maintenance medication). On the other hand, methadone and buprenorphine are common transient medications being used during these detoxification treatments, which can be considered as a further main limitation (selection bias). As this study addressed current trends of psychoactive substance abuse (e.g., synthetic cannabinoids, opioid analgesics, gabapentinoids) and provides robust evaluations at least of a core group of psychoactive substances comprising traditional illicit psychoactive substances, cannabis and nicotine, the results of this study can be valuable to the psychoeducation of addicted individuals and to current restriction/legalization debates. Furthermore, we found further evidence of a special bias (“attraction bias”) of substance-addicted persons in the self-evaluation of harms and benefits of psychoactive substance uses as well as a “treatment bias” of addiction medicine experts assessing harmful psychoactive substances, less harmful as they actually are, as becoming particularly apparent here in the case of methadone.

## Conclusion

This study provides the assessments of users and addiction medicine experts with respect to the harms and benefits of various psychoactive substances, including traditional illicit psychoactive substances, cannabis, alcohol, and nicotine. Also, some modern psychoactive substance use trends related to synthetic cannabinoids and gabapentinoids (Supplementary material S5) were considered. Both, experts and users, ranked the harms arising from the use of alcohol or benzodiazepines (usually unregulated substances) higher than the harms arising from the use of methadone, cannabis or psychotropic mushrooms (regulated by most Western narcotic acts). Thus, also in our study the rankings were not congruent with the narcotics acts of Western nations (as we have hypothesized). The results could be valuable to the psychoeducation of addicted individuals and to current restriction/legalization debates, especially in the Western EU. Comparing the users’ and experts’ substance harm and benefit assessments pointed to a special bias of addicted persons (“attraction bias”) and a “treatment bias” of addiction medicine experts.

## Data availability statement

The original contributions presented in this study are included in the article/Supplementary material, further inquiries can be directed to the corresponding author.

## Ethics statement

The studies involving human participants were reviewed and approved by Medical Faculty of the University of Duisburg-Essen, Essen, Germany. The patients/participants provided their written informed consent to participate in this study.

## Author contributions

UB: conception and design and drafting the manuscript. MS: analysis of the data. A-KK: collection of the data. All authors contributed to the interpretation of data and revising it critically for important intellectual content.
