# Supplementary material for: Differences between users’ and addiction medicine experts’ harm and benefit assessments of licit and illicit psychoactive drugs: Input for psychoeducation and legalization/restriction debates
Source: Front Psychiatry. 2022 Nov 16;13:1041762. doi: 10.3389/fpsyt.2022.1041762 (PMC9709475; doi:10.3389/fpsyt.2022.1041762)
Supplement: Supplementary file 1 [file Data_Sheet_1.PDF]

Supplementary Material

Differences Between Users´ and Addiction Medicine Experts´ Harm and Benefit Assessments of Licit and Illicit Psychoactive Drugs – Input for Psychoeducation and Legalization/Restriction Debates

Udo Bonnet,[1,2,*] Michael Specka, Ann-Kristin Kanti,[3] Norbert Scherbaum[2]

[1]Department of Psychiatry, Psychotherapy and Psychosomatic Medicine, Evangelisches Krankenhaus Castrop-Rauxel, Academic Teaching Hospital of the University of Duisburg-Essen, Castrop-Rauxel, Germany,

[2]LVR-Hospital Essen, Department of Psychiatry and Psychotherapy, Faculty of Medicine, University of Duisburg-Essen, Essen, Germany

[3]Department of Internal Medicine, Evangelisches Krankenhaus Castrop-Rauxel, Academic Teaching Hospital of the University of Duisburg-Essen, Castrop-Rauxel, Germany

*Correspondence: Udo Bonnet, Email: udo.bonnet@uni-due.de

S2. Methods

A

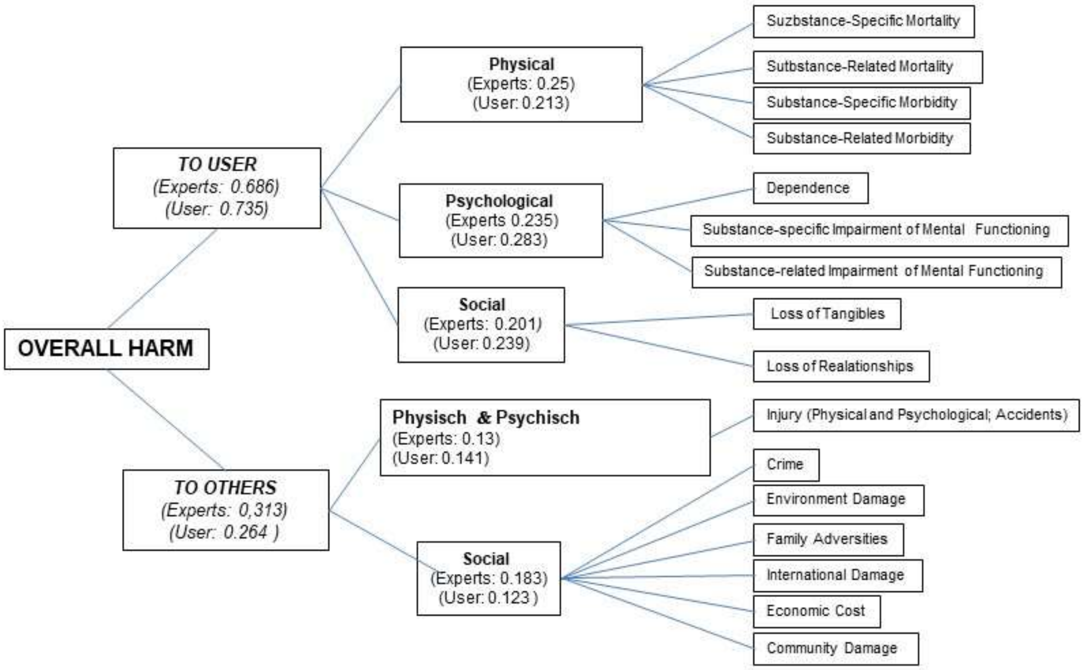

B

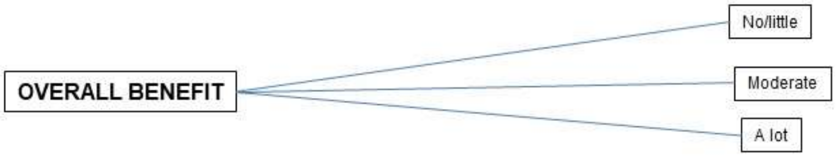

Supplementary Figure S1: Evaluation criteria: Structure of the questionnaire and its evaluation. A: Substance harm rating: for each substance; in a first step (*first survey*), per 5-point scale (from “not harmful” to “extremely harmful”) a sum for every 5 physical, psychological and social dimension (bold letters) being assigned to harms to users and harms to others was determined by the both cohorts 1 of the experts and users. These dimensions had been defined by the 16 criteria usually analysed in studies of this type (all boxes on the right, (1-3)). In a second step (*second survey*), these 5 dimensions were weighted according to their relative harm-relevance for addictive agents (in brackets) by the cohorts 2 of the experts and users. The result of step 1 for every dimension of a substance was multiplied with the weight of this dimension (*in brackets*), separately for the experts and users. The results of all 5 dimensions were summed up to the overall harm of the substance under study, also separately for the experts and users. The calculation of the overall harm of the substances is exemplarily shown below; see Supplementary Material S.2.2. B: Experts and user rankings were expanded by overall benefit ratings of each substance under investigation.

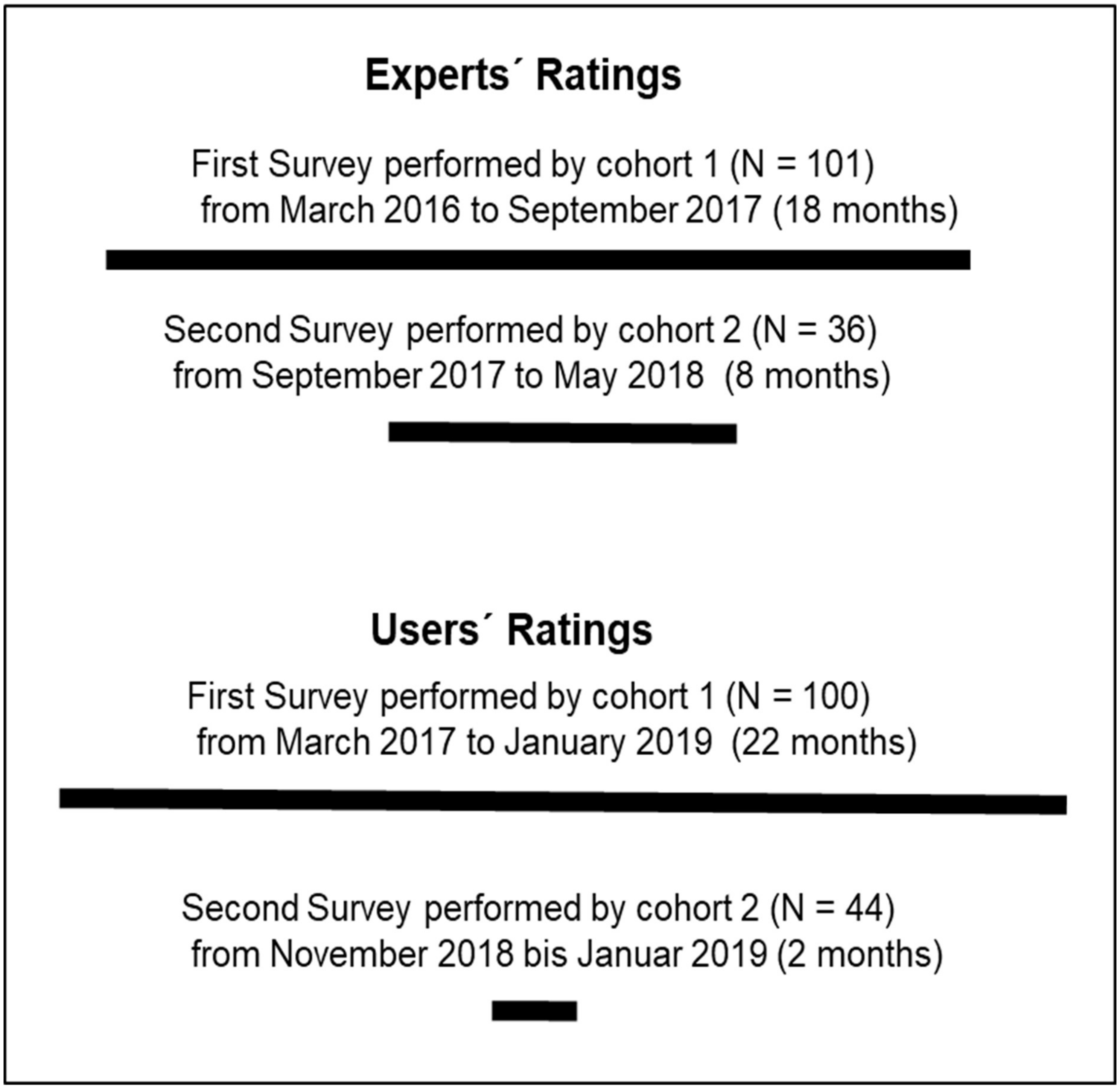

Supplementary Figure S2: Timing of the Surveys. Data collection for expert-cohort 1: the questionnaire was returned by 122 physicians and from those 101 were evaluated since 21 experts did not meet the inclusion criteria (4). Data collection for user-cohort 1 was stopped after 100 interviews had been completed. To achieve this sample size, 117 patients had to be screened. Of these, 6 refused to participate, 3 withdrew from the interview after having given informed consent, and 8 had a MMST level < 25 points.

S2.1. Characteristics of the Ruhr Area and Regulation of Cannabis

The Ruhr Area is a polycentric urban area in North Rhine-Westphalia (NRW), Germany. With a population density of 2800/km^2 and a population of over 5 million people (2017), the Ruhr Area is the largest urban area in Germany and the 5th largest in Europe. It includes several large cities (e.g. Dortmund, Essen, Duisburg, Bochum, Gelsenkirchen) and is bordered by the rivers Ruhr to the south, Lippe to the north, and Rhine to the west. To the east, it is bordered by the Sauerland, a low mountain range.

A representative survey conducted at 2018 revealed that within NRW, about 10% of its 11 million residents aged 18–64 years exhibited risky alcohol use (>12 g alcohol daily in women, >24 g in men) (5). In addition, the following 12 month prevalence rates (in %) for illegal drugs were found: cannabis (6.5%), cocaine (1.6%), amphetamines/methamphetamine (1.1%), MDMA/ecstasy (0.8%), novel psychoactive substances (0.7%), heroin (0.6%). At 2018, approximately 1.2% of the NRW-population aged 18–64 years were found to be substance dependent (according to DSM-IV) on one or several illicit drugs (including 1.1% for cannabis); and 0.4% of the population show substance misuse. These findings were representative for urban regions of the whole country (5).

In NRW (with the Ruhr Area as its sociocultural core zone), addiction treatment services are distributed relatively dense. As in Germany in general, inpatient treatment services for substance-related disorders mainly include (i), a qualified detoxification treatment, carried out in psychiatric hospitals in charge of a defined region and lasting up to 21 days; and (ii), a rehabilitation treatment with a focus on psychotherapy and professional rehabilitation lasting up to 12 weeks.

At the ward for qualified detoxification treatment of the Evangelisches Krankenhaus Castrop-Rauxel (Castrop-Rauxel is a small city directly adjacent to Dortmund), 75% of the user-raters of this survey were recruited. The remaining 25% of user-raters were recruited from the the salus Klinik Castrop-Rauxel, a rehab clinic which is specialized in the treatment of substance addicted young adults.

At the time of the study, the prescription and administration of medical cannabis was legal in Germany in contrast to recreational cannabis use.

S2.2. Overall harm calculation -exemplarily for the user cohort:

Using the weights determined by user cohort 2 (Table 1, Supplementary Figure 1), the average overall harm was calculated as follows:

Overall harm = Physical harm to user x 0.213 + Psychological harm to user x 0.283 + Social harm to the user x 0.239 + Physical and psychological harm to others x 0.141 + Social harm to others x 0.123.

S3. Results

Supplementary Tables

Supplementary Table S1: Users´ overall harm assessment and their experience with a corresponding substance (underlying Figure 5)

	Level of experience with an assessed substance						
	moderate			a lot			
	Overall Harm			*Overall Harm*			p
	**Mean**	**SD**	**n**	**Mean**	**SD**	**n**	
Alcohol	1,98	0,87	15	2,23	0,68	49	0.23
Cannabis	1,48	0,91	10	1,56	0,78	73	0.74
Amphetamines	2,56	0,61	21	2,39	0,76	36	0.40
Ecstasy	1,69	0,80	18	1,83	0,98	25	0.63
LSD	1,45	0,88	14	1,47	0,84	12	0.94
Methadon	1,73	0,93	14	1,66	0,89	41	0.79
Buprenorphine	1,22	0,35	7	1,13	0,49	11	0.69
Cocaine	2,42	0,74	27	2,79	0,83	45	0.63
Psychotropic Mushrooms	0,95	1,17	11	1,26	0,74	9	0.50
Benzodiazepines	2,09	1,00	12	2,00	0,78	25	0.75

*p < 0.01

Supplementary Table S2: Benefit-Harm Ratio **(**underlying Figure 9**)**

	Experts			***User***		
	% no/little benefit	Overall harm	n	% no/little benefit	Overall harm	**n**
**Alcohol**	44,6	2,7	101	33,8	2,2	68
**Amphet.**	59,4	2,4	102	35,1	2,5	57
Buprenorph.	5	1,1	102	14,3	1,2	21
BZD	8,9	1,9	102	23,7	2	38
**Cannabis**	26,7	1,9	102	20,2	1,5	84
Codeine	22,4	1,5	99	27,3	2,2	11
Crack	89,7	3,2	98	16,7	2,8	12
Ecstasy	67,3	2	102	25	1,8	44
Gabap.	7,2	1,1	98	14,3	0,5	7
**Heroin**	60,4	3,1	102	27,7	3,1	65
Ketamine	34,4	2	90	11,1	0,9	9
**Cocaine**	69,3	2,7	102	36,1	2,6	72
LSD	67,3	1,9	99	22,2	1,5	27
**Methadone**	3	1,3	101	17,5	1,7	57
**Nicotine**	74	1,8	101	42,9	1,6	98
Opioid, Analgesics	5,1	1,6	100	28,6	1,9	7
Psychotr. Mushr.	73,1	1,9	93	21,7	1,2	23
Synth. Canna.	75	2,2	92	56	2,2	25
Tilidine	6,1	1,5	99	54,5	1,9	11

Core group in bold

Supplementary Figures

S3.1. Assessment of the average substance harm to users in the 5 separate health and social dimensions

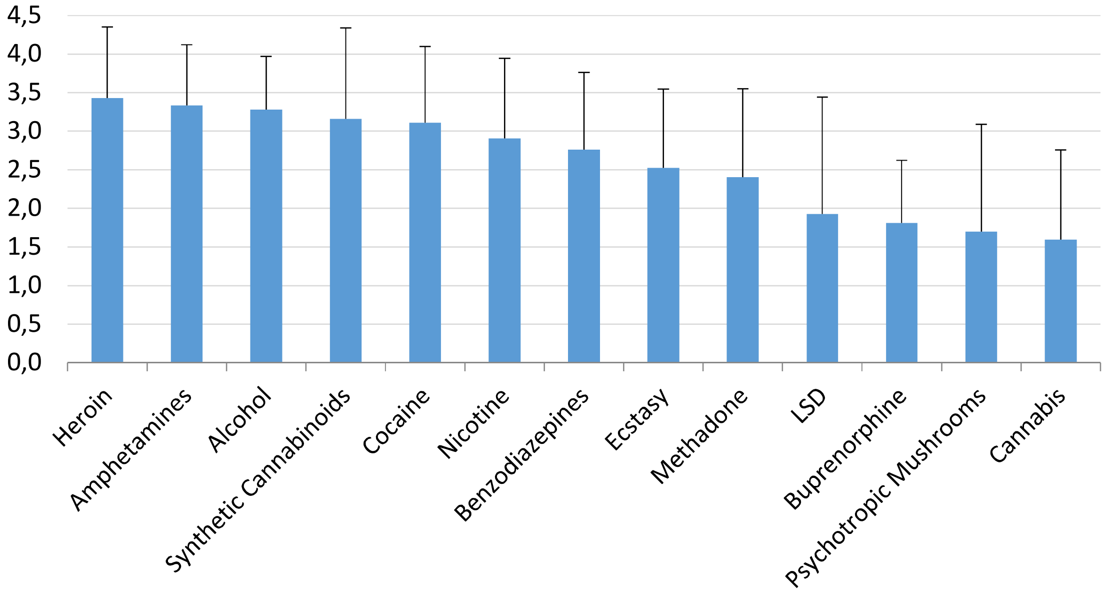

Supplementary Figure S3: Mean (SD) of the 19 evaluated substances in the dimension "**physical harm to users"** on a scale from 0 "not harmful" to 4 "extremely harmful**.**

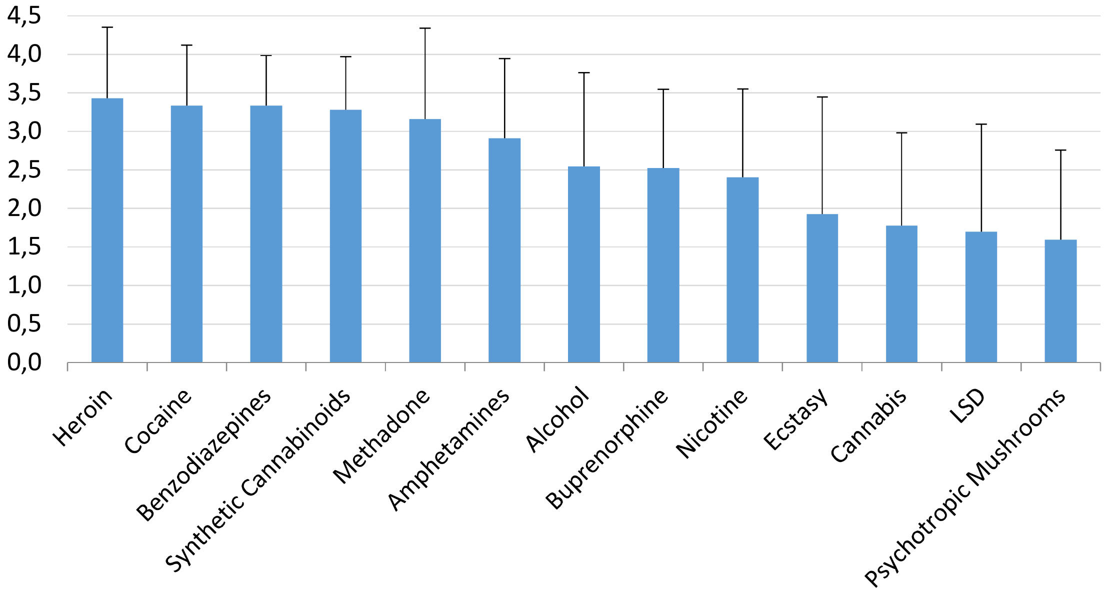

Supplementary Figure S4: Mean (SD) of 13 substances in the dimension **psychological harm to users** on a scale from 0 "not harmful" to 4 "extremely harmful.

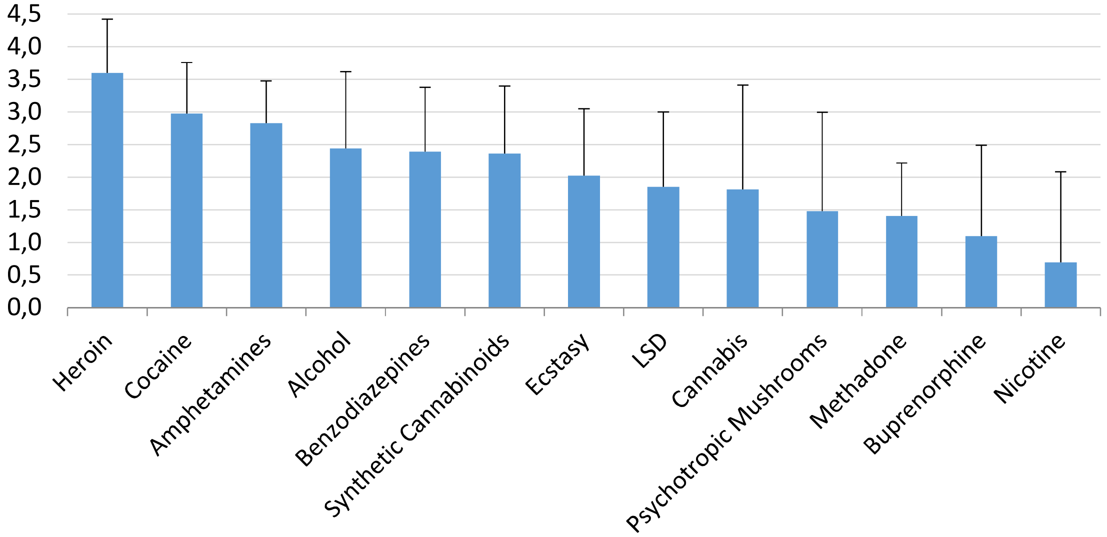

Supplementary Figure S5: Mean (SD) of the evaluated 19 substances in the dimension **social harm to users** on a scale from 0 "not harmful" to 4 "extremely harmful.

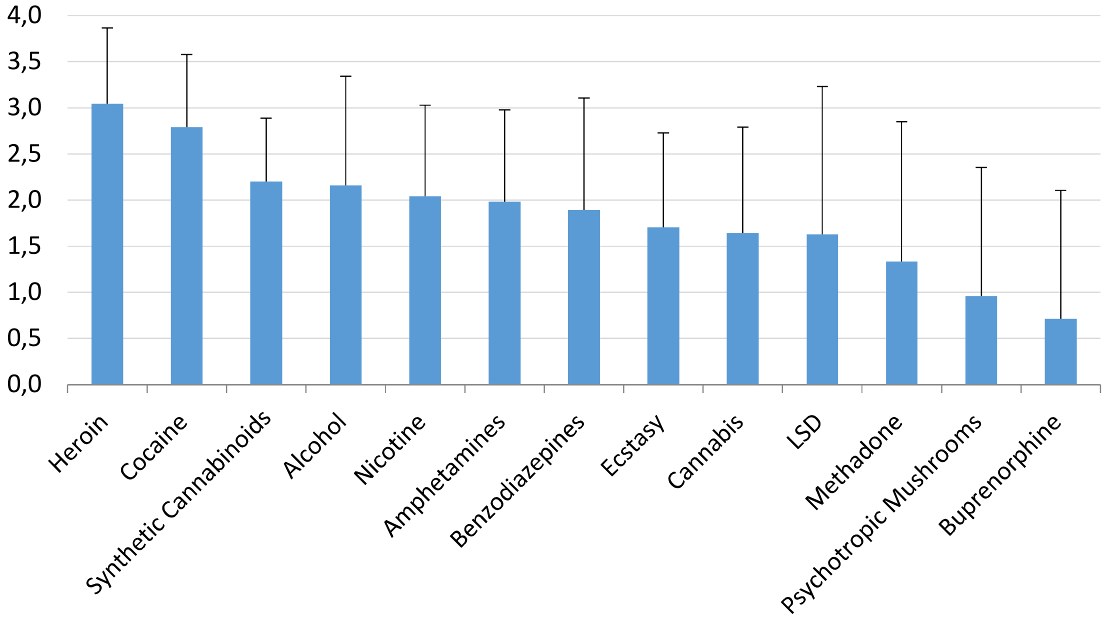

Supplementary Figure S6: Mean (SD) of the evaluated 19 substances in the dimension **physical & psychological harm to others** on a scale from 0 "not harmful" to 4 "extremely harmful.

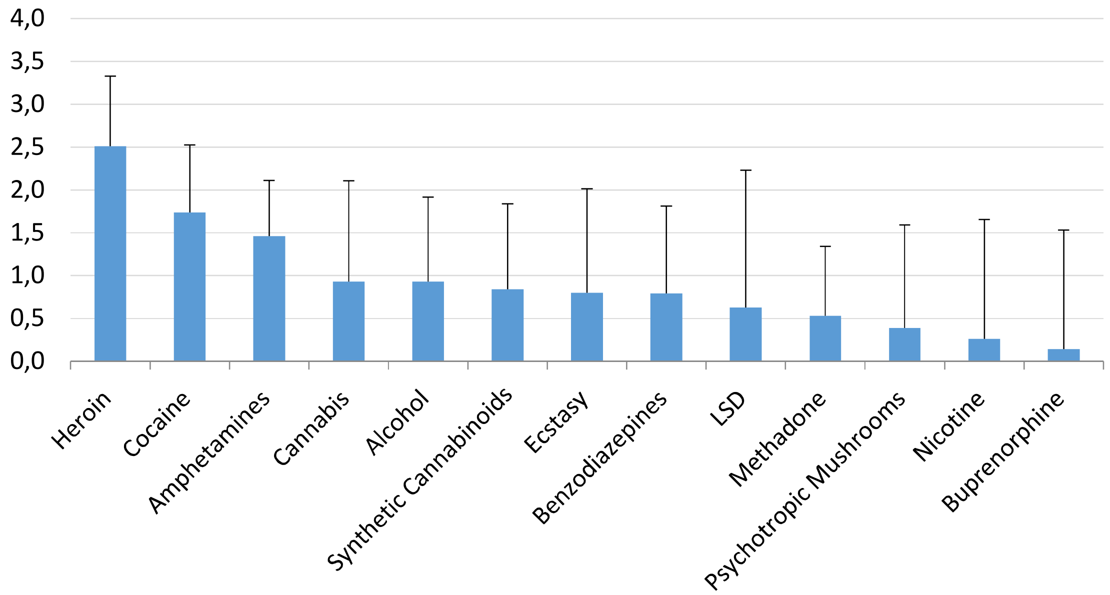

Supplementary Figure S7: Mean (SD) of the evaluated 19 substances in the dimension **social harm to others** on a scale from 0 "not harmful" to 4 "extremely harmful.

S3.2. No relevant differences between the user ratings of inpatients treated in an acute and rehab hospital

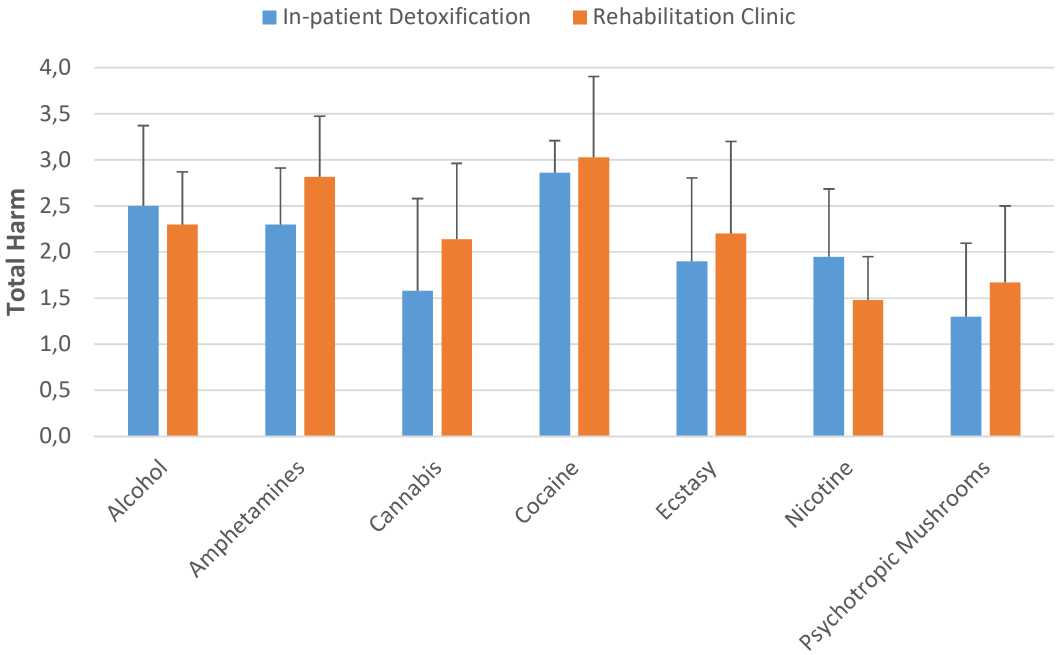

Supplementary Figure S8: Overall harm ratings of users from acute/detoxification ward (n =75) versus rehab clinic (n = 25). Evaluated were all substances with minimum of 6 ratings in each group. Alcohol 59 vs 9, Amphetamines 40 vs 17, Cannabis 61 vs 23, Cocaine 61 vs 11, Ecstasy 32 vs 12, Nicotine 73 vs 25, Mushrooms 17 vs. 6. * p < 0.01.

S3.3. Comparison between the User- and Expert-Ratings

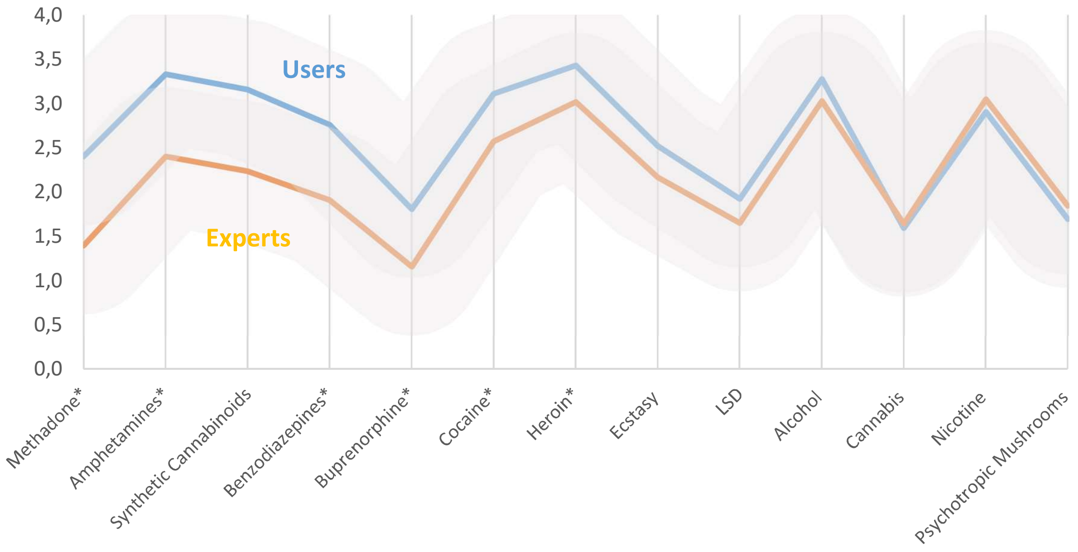

Supplementary Figure S9: Average **physical harm to users** as rated by users versus experts. * = p < 0.01.

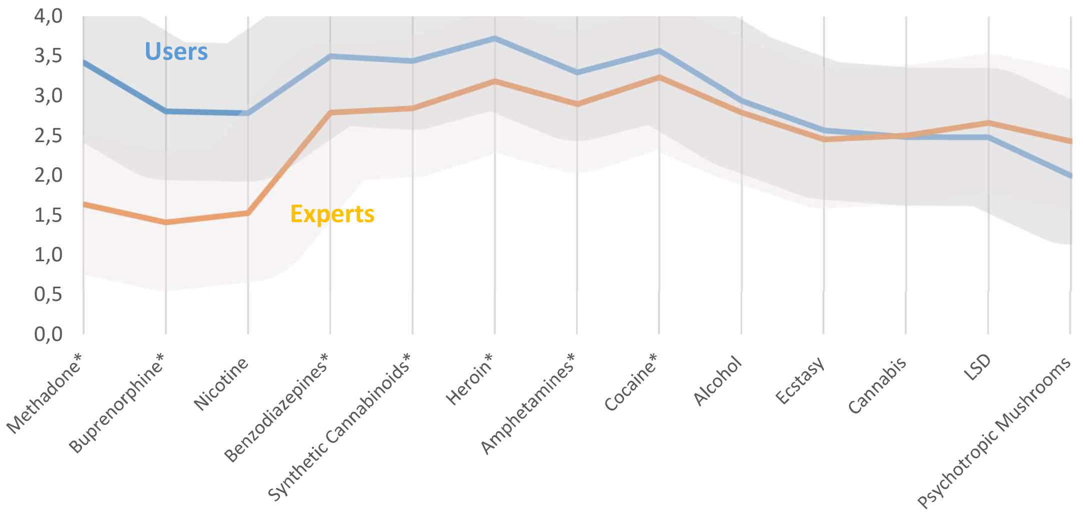

Supplementary Figure S10 Average **psychological harm to users** as rated by users versus experts. * = p < 0.01.

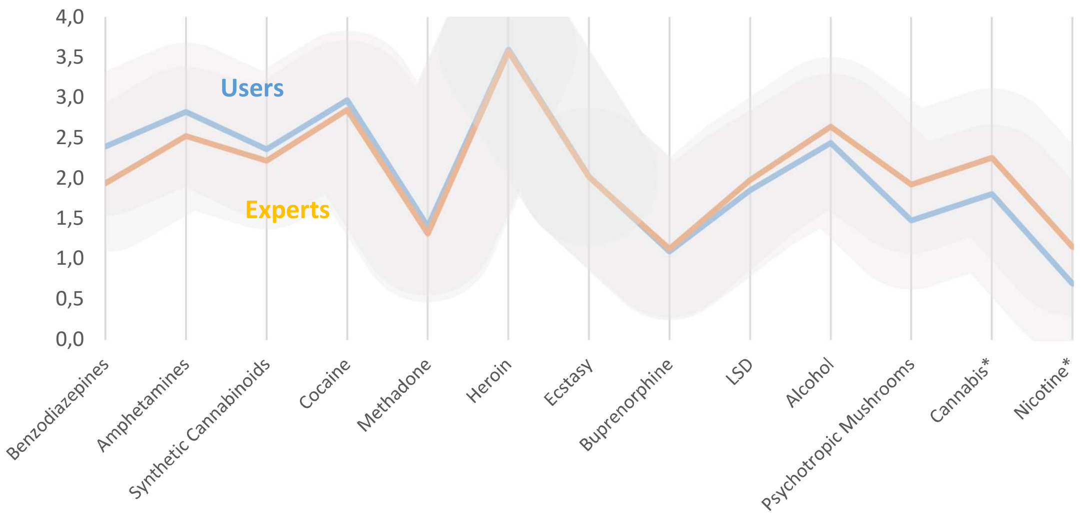

Supplementary Figure S11 Average **social harm to users** as rated by users versus experts* = p < 0.01.

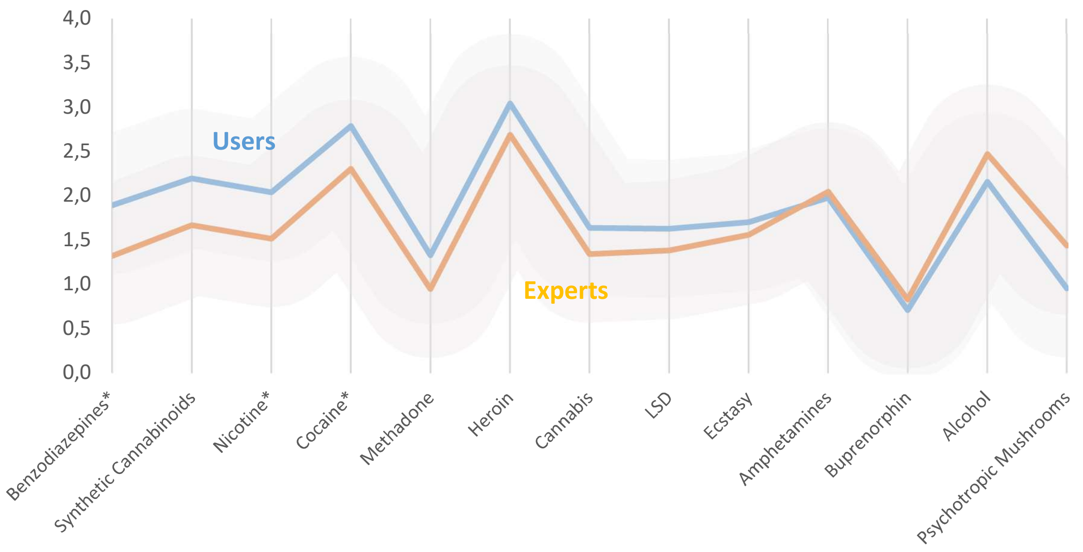

Supplementary Figure S12: Average **physical and psychological harm to others** as rated by users versus experts. * = p < 0.01.

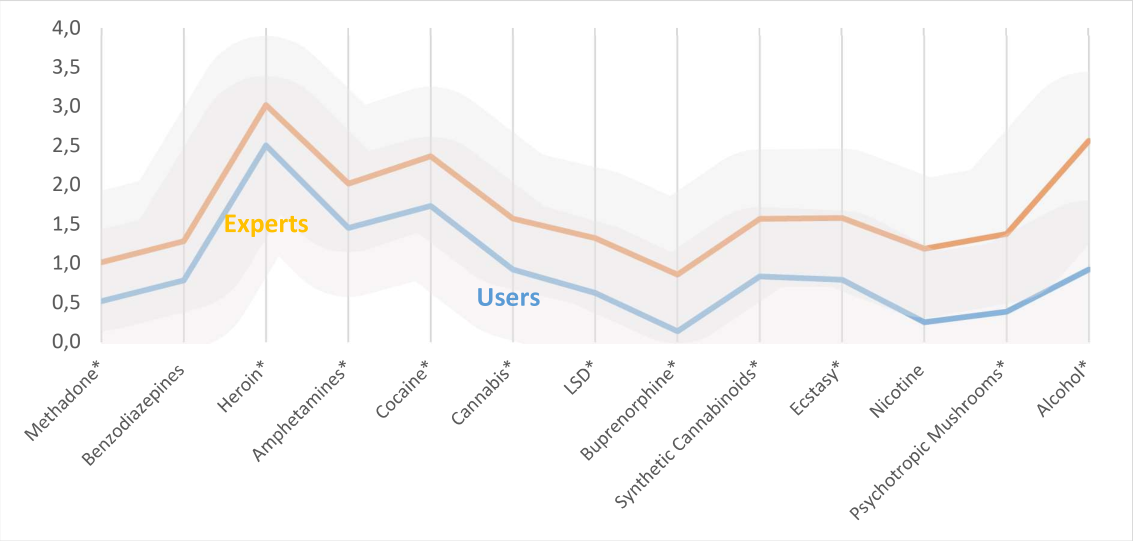

Supplementary Figure S13: Average **social harm to others** as rated by users versus experts* = p < 0.01.

S3.4. Average Overall Benefit/Utility

Comparison between overall benefit ratings of users and experts

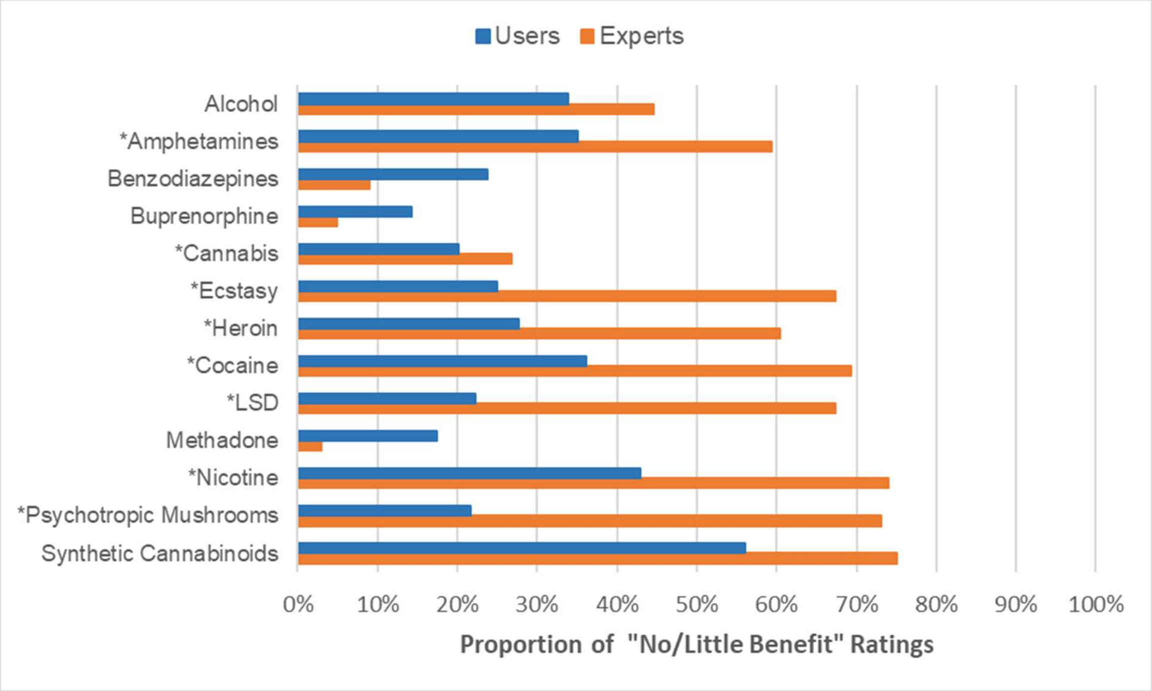

Supplementary Figure S14: Proportion of “no”/”little benefit” ratings made by users versus experts. * = p < 0.01. Supplementary Figure S22 shows also the ratings for the excluded substances.

S4. Consideration of all 33 chosen substances

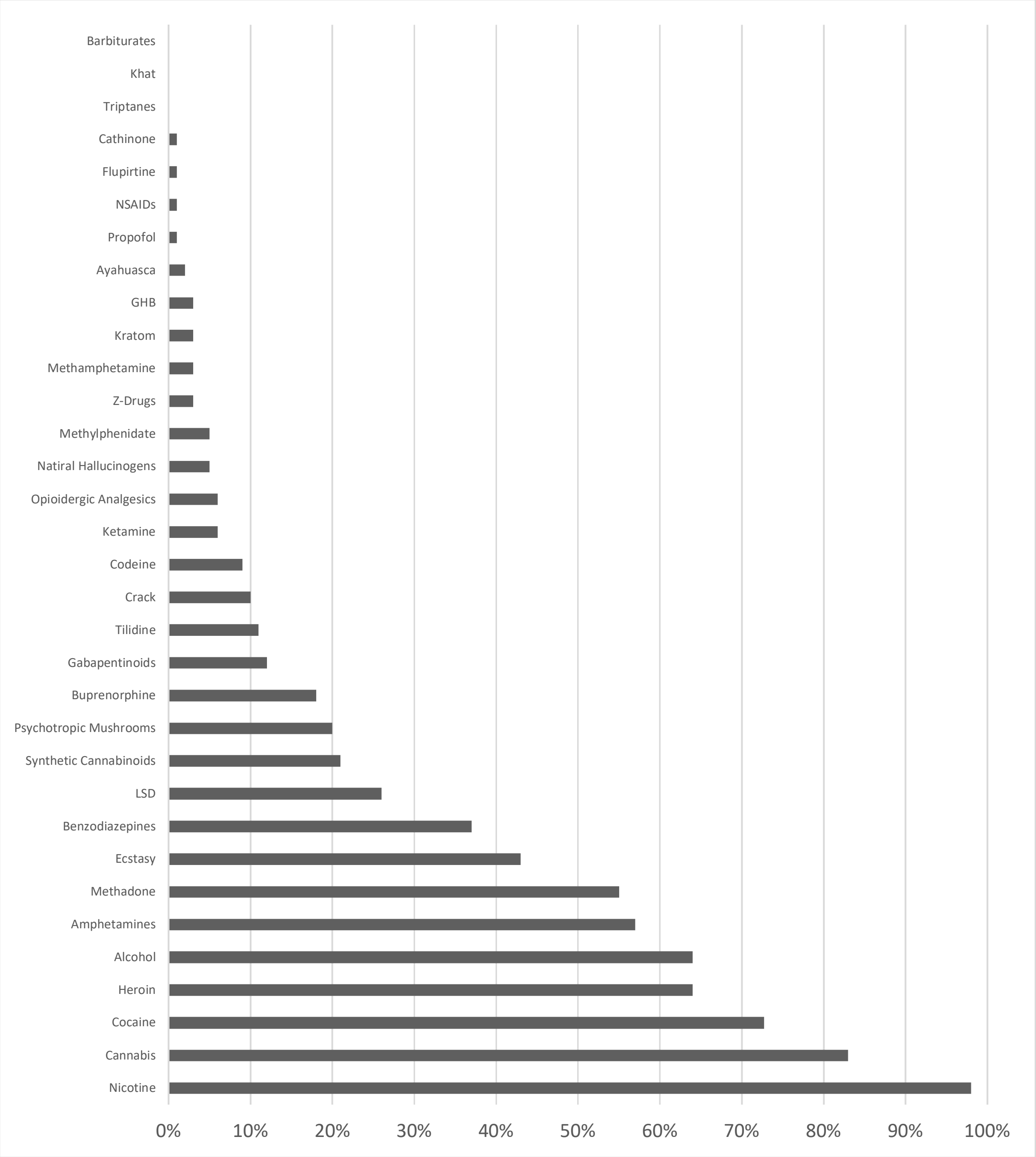

Supplementary Figure S15: Frequency of assessments made per substance by user cohort 1. "Methadone" (covered both methadone and L-polamidon); "LSD" covered also mescaline and "tilidine" also tramadol. Extended version of Figure 1.

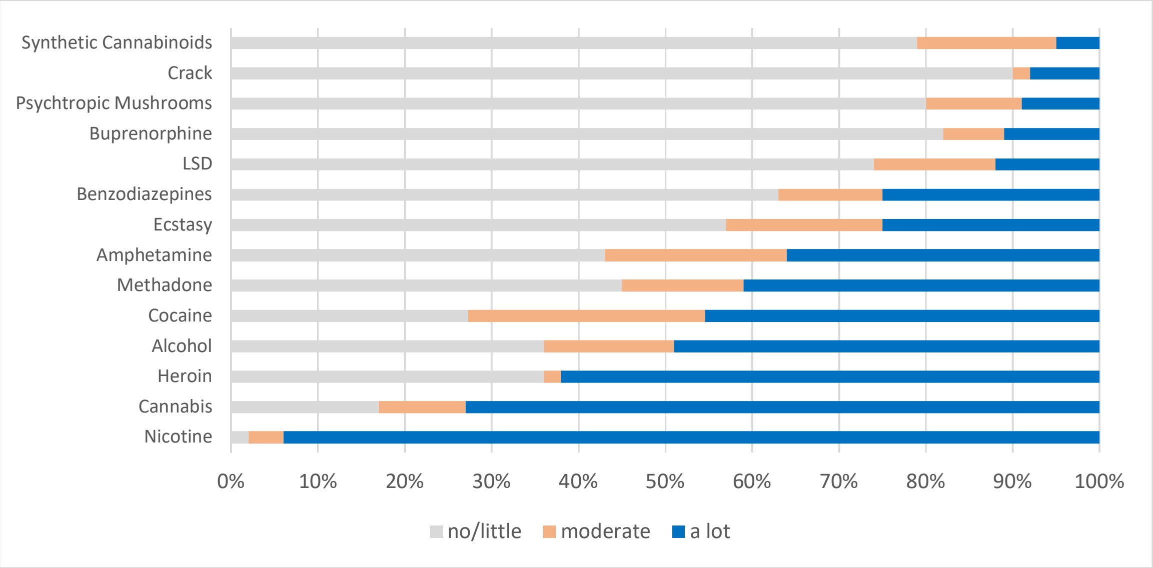

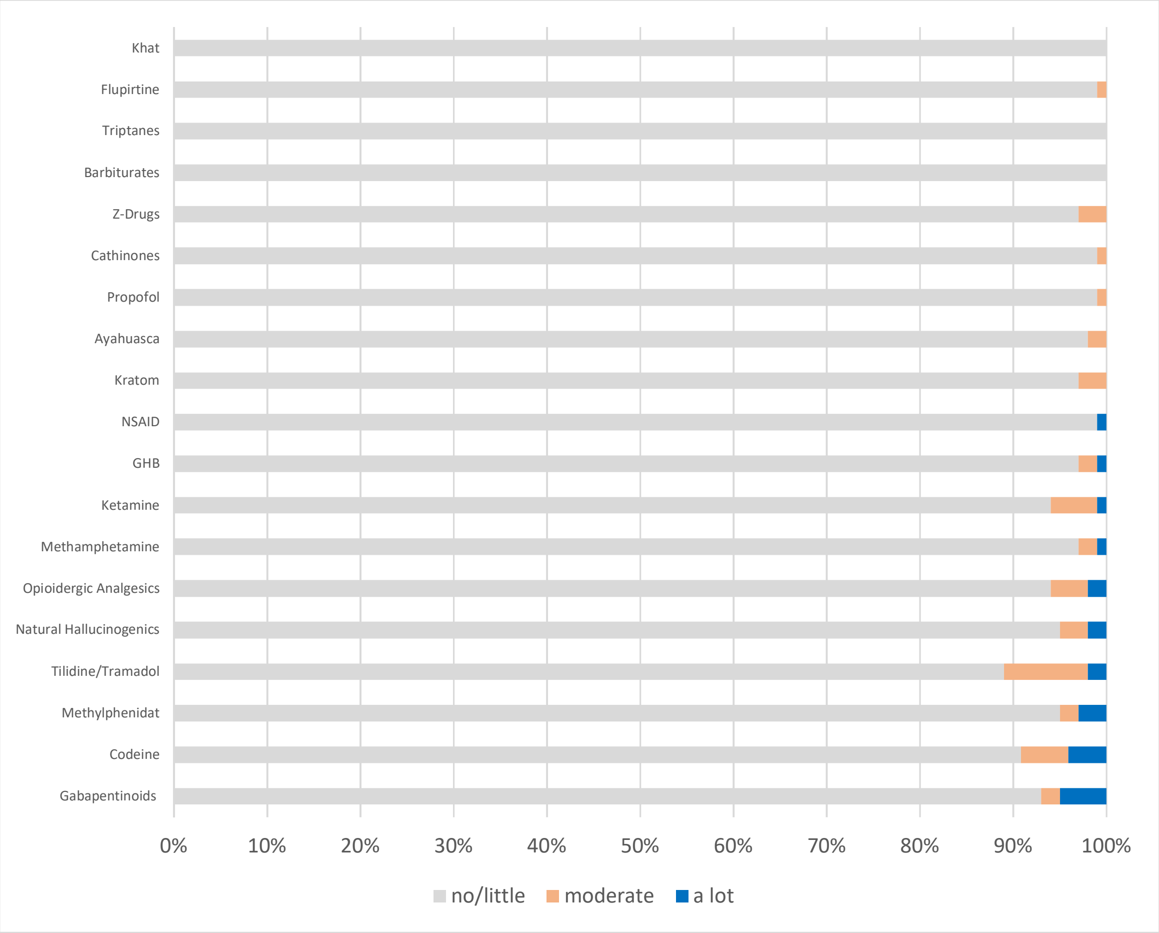

Supplementary Figure 16: Users´ experience regarding the 33 substances in cohort 1. Extended version of Figure 2.

S5. Consideration of all substances with more than 5 ratings

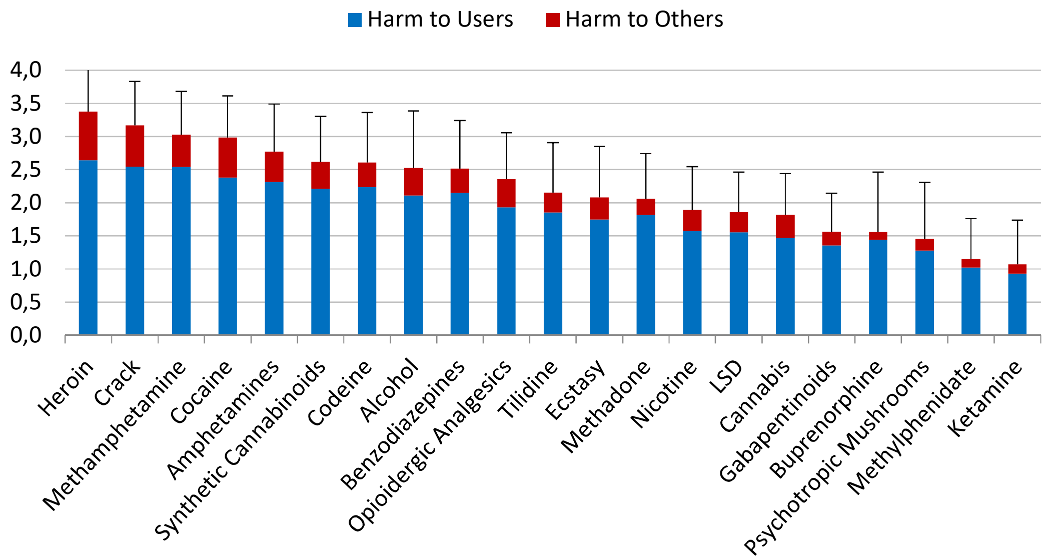

Supplementary Figure S17: Mean (SD) **overall harm** of 21 evaluated psychoactive substances (extension of Figure 3, rated by the users (cohort 1 x weights of cohort 2).

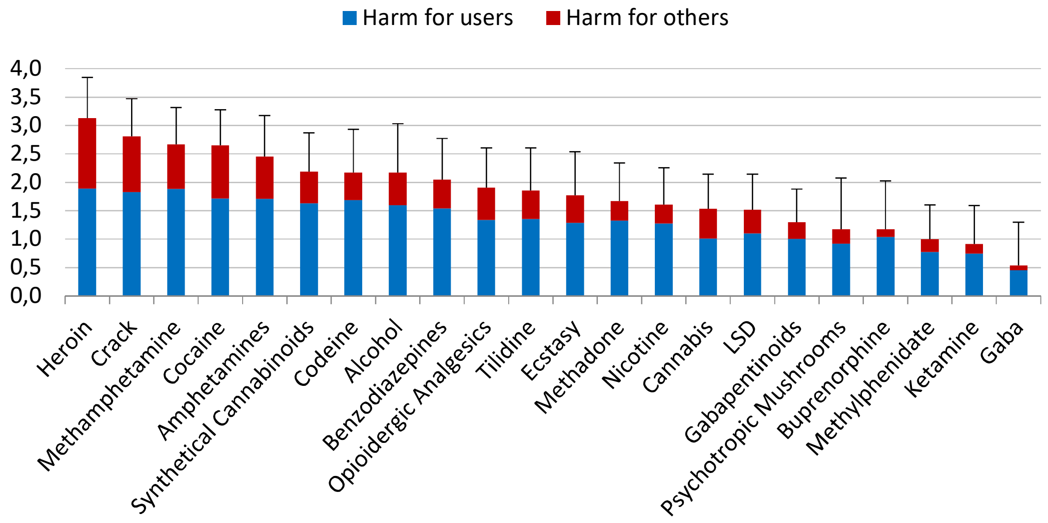

Supplementary Figure S18 : Sensitivity Test - Mean (SD**) overall harm** of 21 evaluated psychoactive substances (extension of Figure 4), rated by the users (cohort 1 x EU-weights, see Table 1, Supplementary Figure 1).

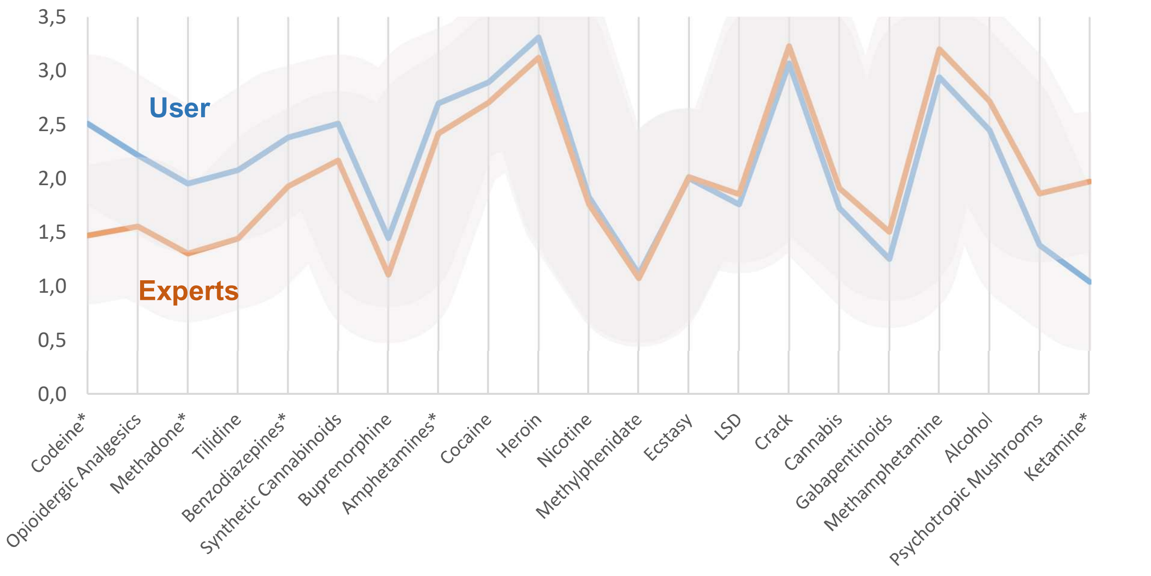

Supplementary Figure S19 : Comparison of the average **overall harm** assessments of the user and experts. 21 evaluated psychoactive substances (extension of Figure 6).

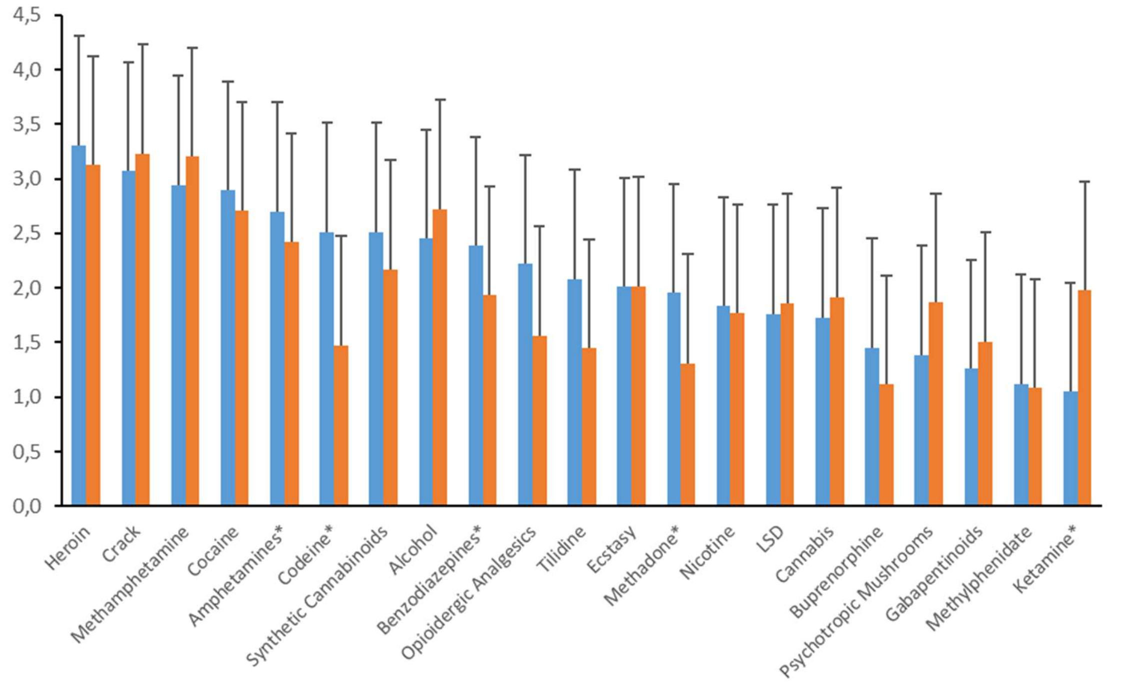

Supplementary Figure S20: Rank order - Comparison between users´ and experts´ average **overall harm ratings.** The relative contribution of the 5 dimensions had been weighted by the cohorts 2 of the user and experts. Especially valid were the ranks of a core group of substances (marked by bold letters) with more than 50% user-assessments per substance (see methods section). See also Figure 6, * = p < 0.01.

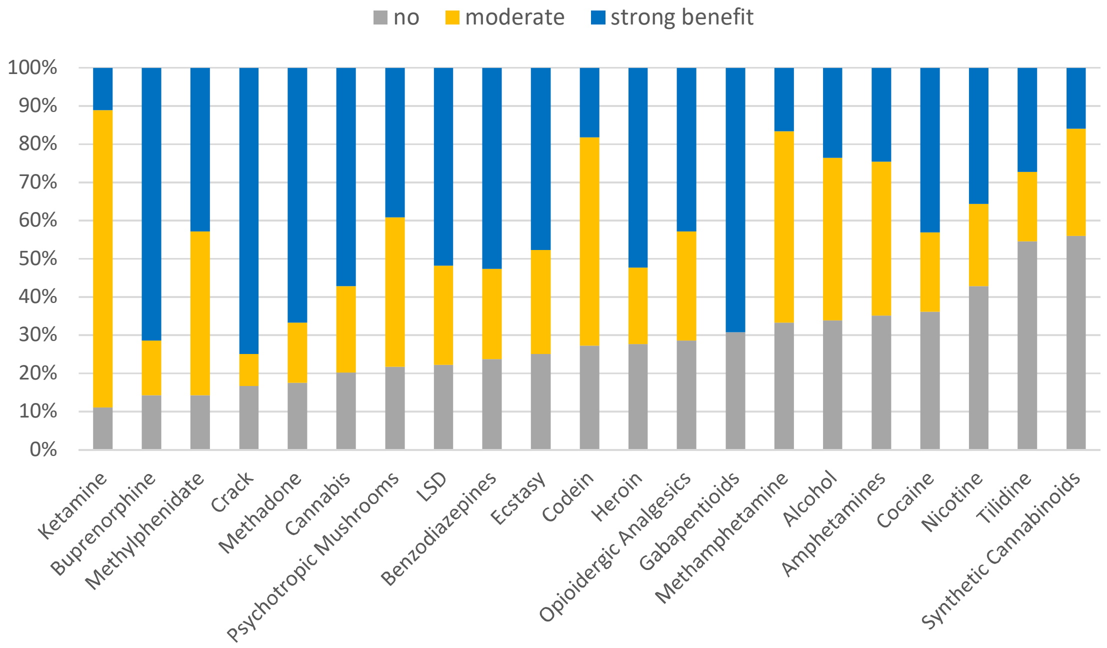

Supplementary Figure S21: **Overall benefit** rating of the users (cohort 1). 21 evaluated psychoactive substances (extension of Figure 7).

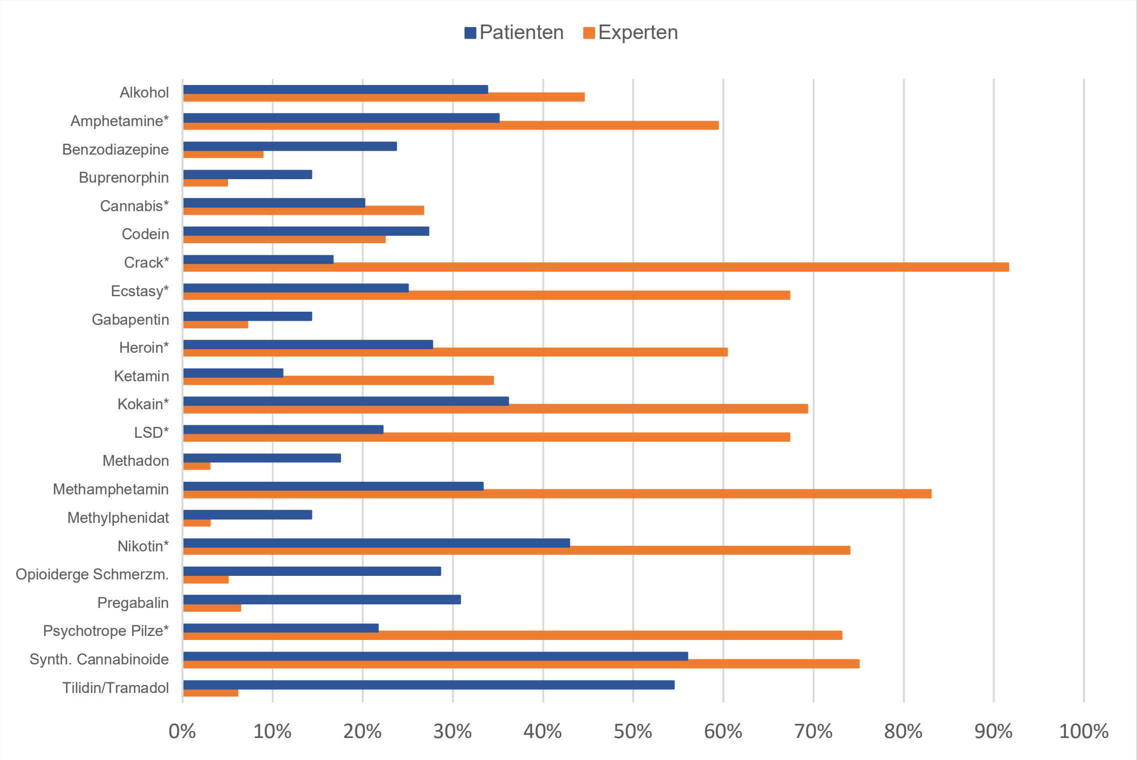

Supplementary Figure S22: Comparison between users´ and experts´ **average benefit** ratings (% "no/little" benefit). 21 evaluated psychoactive substances (extension of Figure S14. * = p < 0.01.

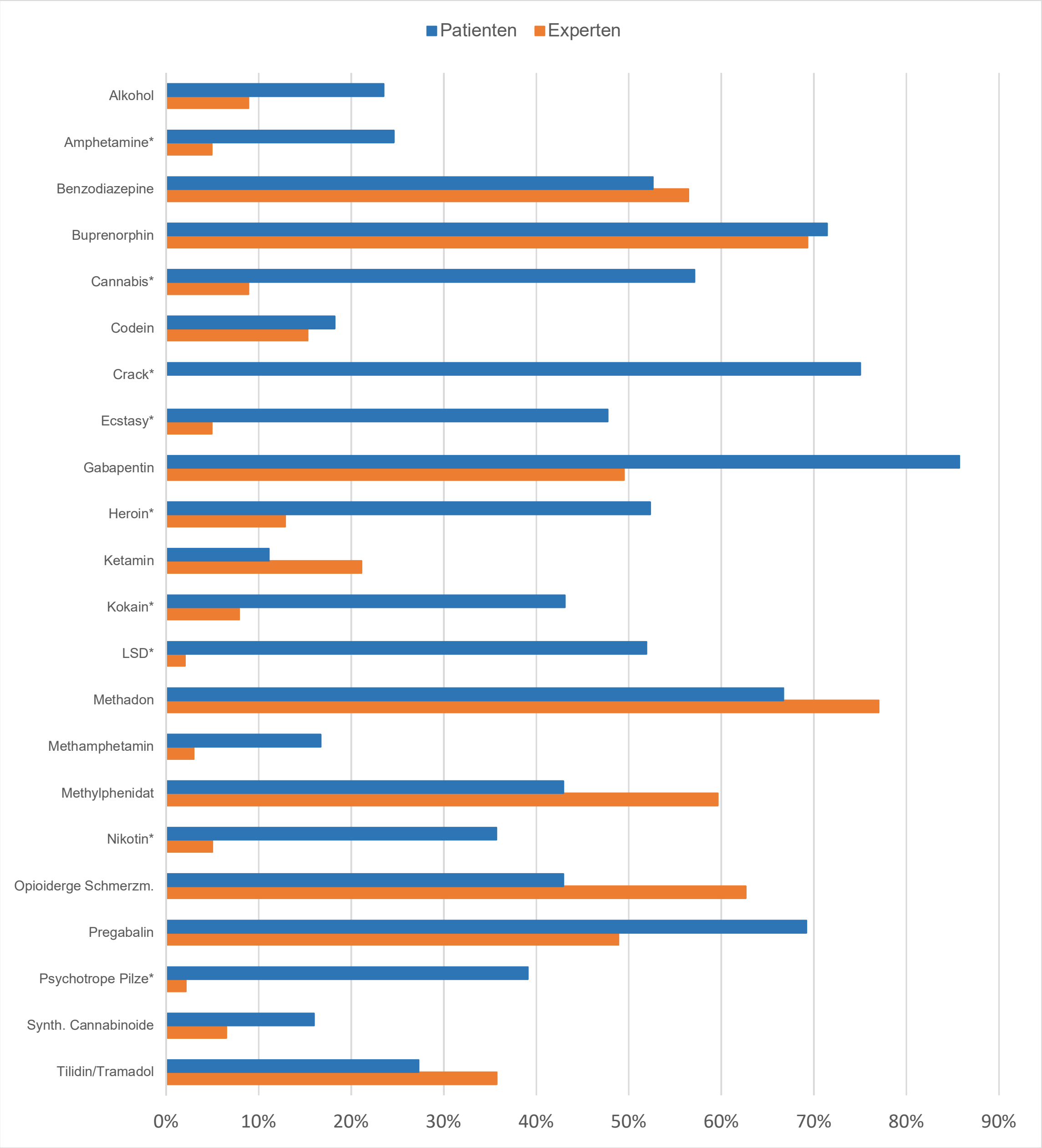

Supplementary Figure S23: Comparison between users´ and experts´ **average benefit** ratings (% “strong” benefit). 21 evaluated psychoactive substances (extension of Figure 8. * = $p < 0.01$.

References

1. Nutt DJ, King LA, Phillips LD. Independent Scientific Committee on Drugs. Drug harms in the UK: a multicriteria decision analysis. Lancet. (2010) 376:1558–65. doi: 10.1016/S0140-6736(10)61462-6.

2. van Amsterdam J, Nutt D, Phillips L, van den Brink W. European rating of drug harms. J Psychopharmacol. (2015) 29: 655–60. doi: 10.1177/0269881115581980.

3. Bonomo Y, Norman A, Biondo S, Bruno R, Daglish M, Dawe S, et al. The Australian drug harms ranking study. J Psychopharmacol. (2019) 33:759–68. doi: 10.1177/0269881119841569.

4. Bonnet U, Specka M, Soyka M, Alberti T, Bender S, Grigoleit T, et al. Ranking the Harm of Psychoactive Drugs Including Prescription Analgesics to Users and Others-A Perspective of German Addiction Medicine Experts. Front Psychiatry. (2020) 11:592199. doi: 10.3389/fpsyt.2020.592199.

5. Seitz N-N, Rauschert C, Atzendorf J, Kraus L. Substanzkonsum und Hinweise auf substanzbezogene Störungen in Berlin, Hessen, Nordrhein-Westfalen, Sachsen und Thüringen. Ergebnisse des Epidemiologischen Suchtsurvey 2018. IFT-Berichte Bd. 190 [Substance Use and Substance Use Disorders in Berlin, Hessen, North Rhine-Westphalia, Saxony and Thuringia. Results of the 2018 Epidemiological Survey of Substance Abuse. IFT-Reports Vol. 190]. Munich: IFT Institut für Therapieforschung. (2020). Available online at:https://www.ift.de/fileadmin/user_upload/esa_laenderberichte/Bd_190_ESA_2018_Bundeslaender.pdf
